# Role of *SFTPD*/miR-335-5p/lnc-HNRNPUL2 axis in colorectal cancer: in silico characterization and clinical validation

**DOI:** 10.1186/s12885-026-15833-6

**Published:** 2026-03-26

**Authors:** Marwa Matboli, Aly Elanwar, Walaa Ibrahim, Mohamed Kamel Hassan, Marwa Ali, Radwa Khaled, Shady Montaser, Sara Keshk, Hadeel Medhat, Marwa M Shafei, Reham Atef Mohamed, Manar Ahmed Fouad

**Affiliations:** 1 Medicinal Biochemistry and Molecular Biology, Faculty of Medicine, Ain Shams University, Cairo, Egypt; 2 Translational and Applied Science Hub (TASH), Faculty of Medicine, Ain Shams University, Cairo, Egypt; 3 Department of General Surgery, Faculty of Medicine, Ain Shams University, Cairo, Egypt; 4 Medical Biochemistry and Molecular Biology, Faculty of Medicine, Kasr Al-Ainy Cairo University, Cairo, Egypt; 5 Biotechnology Program, Zoology Department, Faculty of Science, Port Said University, Port Said, Egypt; 6 Center for Genomics, Helmi Institute, Zewail City for Science and Technology, Giza, Egypt; 7 Pathology Department, Faculty of Oral and Dental medicine, Misr International University, Cairo, Egypt; 8 Pharmacology & Toxicology, Faculty of Pharmacy and Drug Technology, Cairo University, Cairo, Egypt; 9 Pharmacy practice department, Faculty of Pharmacy and drug technology, Egyptian Chinese University, Cairo, Egypt

**Keywords:** Colorectal cancer, Bioinformatics, Diagnosis, SiRNA, MiRNA, lncRNA

## Abstract

**Background:**

The lnc-RNA, miRNA, and mRNA networks have been extensively studied for cancer regulation. Furthermore, ceRNA network analysis has proved as an effective candidate for identifying potential diagnostic biomarkers in a variety of cancers. In this study, we described a novel axis of serum Surfactant protein D (*SFTPD*)/miR-335-5p/lnc-HNRNPUL2 proposed by bioinformatics analysis and validated in a pilot human study of CRC versus control & in vitro assay to explore its role in the CRC pathogenesis.

**Methods:**

Real-time PCR and ELISA were used to validate the expression of this axis panel in serum samples from 81 CRC patients, 41 patients with benign tumors, and 30 healthy controls. Its expression was also confirmed in the CRC cell line HT29.

**Results:**

Lnc-HNRNPUL2 and TGF-β expression levels were significantly upregulted in the sera of CRC patients compared to controls, along with a concurrent decrease in the expression of miR-335-5p and *SFTPD* mRNA in the CRC group relative to both benign and healthy control groups Interestingly, lnc-HNRNPUL2 and hsa-miR-335-5p had significant predictive values for CRC diagnosis due to their high sensitivities and specificities (AUC = 0.901 and 0.877, respectively). In addition, we silenced lnc-HNRNPUL2 in HT29 cells, which resulted in a significant decrease in cell count and viability, as well as a restoration of normal panel expression.

**Conclusion:**

Together, these findings point to an oncogenic role for lnc-HNRNPUL2 in CRC, which acts via miR-335-5p suppression, followed by a decrease in *SFTPD* mRNA that is involved in immune surveillance against several cancers. The *SFTPD*/miR-335-5p/lnc-HNRNPUL2 axis shows high diagnostic accuracy for CRC detection. Low miR-335-5p expression was significantly associated with metastasis in Cox regression analysis, but longer follow-up is needed to establish prognostic value.

**Supplementary Information:**

The online version contains supplementary material available at 10.1186/s12885-026-15833-6.

## Introduction

Colorectal cancer (CRC) is the third most common and the second most fatal cancer worldwide [1], with about 900,000 deaths estimated in 2020 [2]. The global incidence of CRC is predicted to be 2.5 million cases in 2035 [3]. In Egypt, the incidence of CRC is 5.1% in males and 4.7% in females, with a high rate of early CRC–35% of 1,600 Egyptian CRC patients were below 40 [4]. Due to a lack of noninvasive and early screening tools, CRC is diagnosed at a late stage when cancer has caused occlusion or metastasis. In most cases, surgery, radiation, and chemotherapy are used as treatments despite being ineffective in eradicating cancer [5].

CRC represents a complex disease involving diverse genetic events, associated with immune response, and the impact of exogenous factors [6]. As a result, it is challenging to understand the complex immune responses in the tumor microenvironment and the genetic and/or epigenetic changes in CRC. Despite widespread clinical use, serum biomarkers such as carcinoembryonic antigen (CEA) and Carbohydrate antigen 19.9 (CA19-9) lack sufficient sensitivity and specificity for early detection and prognostic stratification in colorectal cancer. Therefore, the identification of novel, minimally invasive molecular biomarkers remains a critical unmet need [7, 8]. One of the hallmarks of cancer is tumor evasion from immune surveillance [9]. The immune system of the body has an anti-tumor effect under normal conditions. However, if the immune surveillance is reduced, malignant tumors may develop through diverse immune-escape mechanisms. Tumor cells and their microenvironment can be altered to escape detection and targeting by the immune system [8].

Surfactant protein D (*SFTPD*) is a collagenous glycoprotein encoded by the *SFTPD* gene from the collectin family [10]. It is an essential innate immune molecule involved in pathogen clearance and inflammation regulation at pulmonary and extrapulmonary sites [11]. It also acts as an immune surveillance molecule against lung and pancreatic cancer [11]. *SFTPD* is a pattern recognition molecule with a potential anticancer innate immune defense molecule. *SFTPD* induces tumor necrosis factor-α (TNF-α) and Fas-mediated pro-apoptotic signaling pathways and activates the caspase cascade, and thus, induces apoptosis in pancreatic ductal adenocarcinoma [12]. It interrupts epidermal growth factor (EGF) signaling & inhibits epithelial-to-mesenchymal transition by inhibiting the transforming growth factor-β (TGF-β) pathway [12].

Long noncoding RNAs (lnc-RNAs) primarily regulate transcription by interacting with other molecules, including microRNAs (miRNAs) and messenger RNAs (mRNAs). The competing endogenous RNA (ceRNA) hypothesis explains these interactions and their impact on protein expression [13]. LncRNAs are involved in both innate and adaptive immunity by regulating the expression of immune response genes at the transcriptional or epigenetic levels in immune cells [14]. They have been identified as essential regulators in cancer immunity [15]; in CRC tissues, the expression of the lnc-RNA SNHG20 was significantly upregulated [16], while that of LINC00485 was significantly downregulated compared with normal tissue [17].

miRNAs, a part of the ncRNA family with an average length of 22 nucleotides, are molecules that regulate cell differentiation, development, apoptosis, immune responses, hematopoiesis, cell death, and proliferation [18]. Numerous miRNAs have been found to be abnormally expressed in CRC, where they regulate various targets [18]. In CRC tissues, the expression of miR-1-3p, miR-133a-3p, miR-133b-3p, and miR-206-3p was lower than in normal tissues; a high level of these miRNAs is associated with a good prognosis [19].

When the TGF-β is overexpressed in the tumor microenvironment, immune surveillance is suppressed, which makes it easier for anti-tumor immune cells to migrate, escape, and become more resistant to attacking the tumor. Although TGF- suppresses the expression of the surfactant proteins SP-A, SP-B, and SP-C in the lungs, its impact on *SFTPD* is poorly understood [20]. Another study explained their linkage that the immune surveillance of *SFTPD* attenuates the TGF-β signaling pathway in pancreatic cancer [21].

Tumor biopsy is the classic strategy in CRC diagnosis [22]. However, due to CRC heterogeneity, liquid biopsies provide better non-invasive, easy access to tumor material shedding into circulation and entering the bloodstream like circulating tumor cells, circulating free DNA and RNA, extracellular vesicles, or proteins that imitate the metastatic environment for a tumor’s invasion and migration [23]. In addition, liquid biopsy unveils disease evolution over time, epigenetic alterations, and escape mutations caused by cancer heterogeneity, and even tumor recurrence [24, 25].

The current pilot clinical study” aims at “hypothesis generation and initial clinical validation.” It is important to note that the primary aim of this study was the clinical validation of a bioinformatically-derived RNA panel as a diagnostic biomarker in CRC patient sera. While the proposed ceRNA network provides a mechanistic hypothesis, direct molecular validation of RNA–RNA interactions falls outside the translational scope of this work and remains a target for future investigation. We used bioinformatics analysis to retrieve an RNA panel (mRNA, miRNA, and lnc-RNA) linked to immune dysregulation in CRC. Furthermore, we incorporated TGF-β into the panel based on its established dual role in CRC: (1) as a validated target of miR-335-5p (as confirmed in miRTarBase), and (2) as a master immunosuppressive cytokine that promotes immune evasion—a process potentially counter-regulated by SFTPD-mediated immune surveillance. This positions TGF-β not merely as an associated factor, but as a functional effector downstream of the ceRNA axis, linking RNA-level dysregulation to the immunosuppressive tumor microenvironment. Followed by assessing its expression in the sera of CRC patients to evaluate the panel’s significance as a novel biomarker panel in the CRC diagnosis. Furthermore, we performed the in vitro validation for the mechanistic relation of the mRNA and the epigenetic regulators (miRNA and lnc-RNA). Here, we propose a new biomarker panel, namely the *SFTPD* panel, for the diagnosis of CRC. Using in silico data analysis, we identified *SFTPD* as a CRC gene linked to innate immunity and immune surveillance. We completed the network panel (*SFTPD*/miR-335-5p/lnc-HNRNPUL2) by identifying lnc-HNRNPUL2, hsa-miR-335-5p, and TGF-β. The utility of this panel was validated by analyzing its expression in CRC patients’ sera and a representative colorectal cancer cell line.

## Materials and methods

### Ethical approval

In the current pilot study, we enrolled a total of 152 individuals from the Ain Shams University Department of Gastrointestinal and Colorectal Surgery randomly from December 2023 to December 2024. Physical examination confirmed that the healthy participants in this study did not have malignancy, severe morbidity, or gastrointestinal infection. All participants provided written consent, and the study followed the guidelines outlined in the Declaration of Helsinki. The study protocol was approved by the Ain Shams Faculty of Medicine Ethics Committee (FWA No: FAMSU R 106/2022).

### Patients and samples

Patients who participated in this study met the following inclusion criteria: ((1) healthy controls had a recently completed normal colonoscopy with no prior history of colorectal cancer (CRC); (2) CRC cases were confirmed by two independent clinical pathologists; (3) participants had not received chemotherapy, radiotherapy, or immunotherapy prior to sample collection; and (4) all participants were adults older than 18 years. Patients who had received chemotherapy, radiotherapy, or immunotherapy, as well as those diagnosed with hereditary non-polyposis CRC or familial adenomatous polyposis, were excluded from the study.

Patients with CRC were diagnosed using the American Cancer Society guidelines. According to TNM staging, the clinical stage of the CRC patients was determined: 63 (77.8%) were in the early stage (stages 0, I, and II) and 18 (22.2%) were in the late stage (stages III & IV). Whereas, Stage I (localized), in which the cancer is limited to the site of origin; Stage II (regional), in which cancer has spread beyond the initial site, frequently to nearby lymph nodes; Stage III (distal), in which cancer has spread more widely; and Stage IV, in which cancer has metastasized to distant organs [26].

Moreover, 41 patients with benign colorectal polyps (adenomatous and hyperplastic polyps) were confirmed by histopathological examination, and follow-up showed no cancer progression during the study. Specifically, the benign group consisted of: Hyperplastic polyps (*n* = 16, 39.0%), Tubular adenomas (*n* = 17, 41.5% and Tubulovillous/villous adenomas (*n* = 8, 19.5%). All patients were treatment-naïve at sampling. In addition, 30 sex-matched healthy controls were enrolled. Healthy control participants were recruited from individuals undergoing routine colonoscopy screening at Ain Shams University Hospital. Inclusion criteria required: (1) a completely normal colonoscopy with no polyps, inflammation, or other abnormalities; (2) no personal history of colorectal cancer or inflammatory bowel disease; (3) no first-degree relatives with CRC; and (4) age between 30 and 70 years. Control participants were frequency-matched to CRC cases by age (± 5 years) and sex. While BMI differed significantly between groups (Table 1), we employed statistical adjustment in relevant analyses to account for this potential confounding factor.

**Table 1:** Demographic and clinicopathological characteristics of the studied groups

Variable	Control *N* = 30	Benign *N* = 41	Malignant *N* = 81	*P*-value
Age (Mean ± SD)	43.8 ± 12.69	50.4 ± 13.7	48.17 ± 11.24	0.084
Sex				0.211
Male	19(63.3%)	23(56.1%)	37(45.7%)	
Female	11(36.7%)	18(43.9%)	44(45.3%)	
BMI	20 (15–30)	21 (20–23)	22 (20–23) ^a, b^	0.006
Mean Rank	89.2	88.01	65.97	
Smoking	6 (20%)	12 (29.3%)	17 (21%)	0.536
Family history	6(20%)	13 (31.7%)	26 (32.1%)	0.437
Colonoscopy				0.00
Negative	30 (100.0%)	0 (0%)	0 (0%)	
Mass	0	4 (9.8%)	48 (59.3%)	
Polyp	0	34 (80.5%)	12 (14.8%)	
Other	0	4 (9.8%)	21 (25.9%)	
Type				0.00
Adenocarcinoma		0 (0%)	65 (80.2%)	
mucinious adenocarcinoma		0 (0%)	13 (16.0%)	
signet ring carcinoma		0 (0%)	3 (3.7%)	
hyperplastic polyp		16 (39.0%)	0 (0%)	
Tubular		17(41.5%)	0 (0%)	
Villous		8(19.5%)	0 (0%)	
Grade				-
1			7(8.6%)	
2			56 (69.1%)	
3			15(18.5%)	
4			3(3.7%)	
Stage				-
0			3(3.7%)	
1			22(27.2%)	
2			38(46.9%)	
3			12(14.8%)	
4			6(7.4%)	
Metastasis			14(17.3%)	
Relapse	-	-	29(35.8%)	-

Data expressed as mean ± SD, median (IQR), or n (%). *P*-values from ANOVA (normal), Kruskal–Wallis (non-normal), or Chi-square (categorical)

Post-hoc: Tukey (normal) or Dunn’s (non-normal). ^a^*P*< 0.05 compared with the control group, ^b^*P*<0.05 compared with the benign group

All blood samples were collected prior to any therapeutic intervention (surgery, chemotherapy, radiotherapy, or immunotherapy) to ensure that biomarker levels reflected the natural disease state without treatment-related confounding. Every patient’s detailed clinical data were available. CA19.9 and CEA were measured in the serum samples by an immunoradiometric technique, according to the manufacturer’s instructions. Venous blood (5 mL) was withdrawn from each participant, centrifuged at 3000 rpm for 10 min, and stored at − 60 °C immediately.

### Bioinformatics in the selection of the RNA-based network

**Fig. 1:**
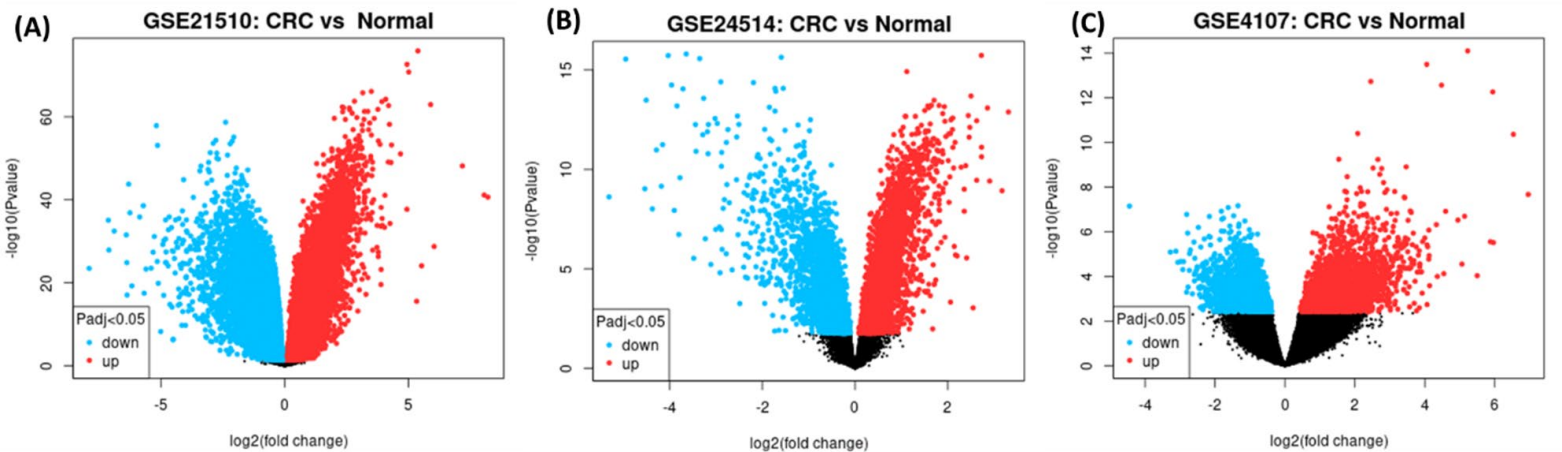
Volcano plots showing DEGs between CRC and normal samples in three independent GEO datasets: (**A**) GSE21510, (**B**) GSE24514, and (**C**) GSE4107. The x-axis represents the log2 fold change (CRC vs. normal), and the y-axis represents −log10 of the adjusted P value. Genes with adjusted P value (Padj) < 0.05 are highlighted: upregulated genes in CRC are shown in red, downregulated genes are shown in blue, and non-significant genes are shown in black

We used the Gene Expression Omnibus (GEO) database (https://www.ncbi.nlm.nih.gov/gds/?term=, assessed December 2025) to find differentially expressed genes (DEGs) in CRC using the following criteria: Organism: *Homo sapiens*, Study type: Expression profiling by array or RNA-seq, Sample type: Primary colorectal cancer tissue vs. adjacent normal/matched normal tissue. Exclusion criteria: Cell lines, metastatic samples, treated samples (chemotherapy/radiation), and studies with fewer than 10 samples per group. Three datasets meeting these criteria were selected: GSE21510: 37 CRC vs. 37 normal, GSE24514: 29 CRC vs. 29 normal, GSE4107: 25 CRC vs. 25 normal (Supplementary Tables S1 & S2) [27–29]. Moreover, these datasets represented independent cohorts from different institutions, as well as provided raw or normalized data for consistent re-analysis. Differential expression was determined using GEO2R (https://www.ncbi.nlm.nih.gov/geo/geo2r/), an interactive web tool that employs the limma R package for analysis. For each dataset, we used the default GEO2R analysis pipeline, which includes: Background correction and normalization as originally applied by the dataset submitters and maintained in GEO’s processed data. Linear modeling via limma to compute moderated t-statistics. *P*-value adjustment using the Benjamini-Hochberg false discovery rate (FDR) method. Probe IDs were mapped to official gene symbols using the platform annotation files provided in GEO. DEG intersection strategy: A gene was considered a “consistently dysregulated candidate” if it met the significance and fold change thresholds (FDR < 0.05, |log₂FC| ≥ 1) in the same direction (up or down) in at least two of the three independent datasets (Fig. 1, Supplementary table S3).

i. Target gene selection

Based on our interest in immune response and inflammatory response regulation pathways, *SFTPD* mRNA was selected for its novelty, differential expression, tissue specificity, and functional relevance to immune surveillance. To validate its relevance in CRC, we used the Expression ATLAS database (https://www.ebi.ac.uk/gxa/home), which confirmed that *SFTPD* is consistently downregulated in colorectal cancer tissues. Transcripts per million (TPM) expression data revealed strong expression in normal colon tissue (intense blue) and reduced expression in CRC samples (light to medium blue), supporting its role as a potential tumor suppressor (Supplementary Figure S1). Functional annotation from the UniProt database (https://www.uniprot.org/) further confirmed *SFTPD’s* involvement in immune response regulation, pathogen clearance, and cancer-related pathways (Supplementary Figure S3).

ii. Selection of epigenetic regulators

To construct a potential ceRNA network centered on *SFTPD*, we identified interacting miRNAs and lnc-RNAs through a multi-database approach. First, candidate miRNAs targeting *SFTPD* were predicted using three independent resources: miRBase, DIANA-TarBase v8, and miRTarBase. From these, miR-335-5p was selected based on (1) high-confidence binding predictions to the *SFTPD* 3′ untranslated region (UTR) across all three databases, (2) experimentally validated interactions documented in DIANA and miRTarBase, and (3) its established roles in cancer and immune regulation. The predicted and validated interaction between miR-335-5p and *SFTPD* is illustrated in Supplementary Figure S3. Second, to assess the biological relevance of miR-335-5p in CRC, KEGG pathway analysis was performed with DIANA-miRPath v3.0, revealing significant enrichment of this miRNA in pathways related to TGF-β signaling, immune response, and colorectal cancer (Supplementary Figure S4). Expression validation using the GeneCards Database confirmed that miR-335-5p is expressed in colon tissue (Supplementary Figure S5), supporting its potential functional role in CRC. Third, to identify lnc-RNAs that could act as competing endogenous RNAs for miR-335-5p, we employed the InCeDB database for miRNA–lnc-RNA interaction prediction and LncBook 2.0 for expression validation in CRC. lnc-HNRNPUL2 (NONHSAT021790, ENST00000540127.1) emerged as a candidate based on (1) a high-confidence predicted interaction with miR-335-5p in InCeDB (Supplementary Figure S6), (2) significant upregulation in CRC tissues according to LncBook expression data (Supplementary Figure S7), and (3) reported involvement in RNA stability and cancer progression. Additionally, the interaction between TGF-β1 and miR-335-5p was validated using miRTarBase, confirming TGF-β1 as a direct target of miR-335-5p (Supplementary Figure S8), consistent with prior studies showing miR-335-5p-mediated regulation of TGF-β signaling. In silico analysis via the Ensembl database further indicated a direct interaction between lnc-HNRNPUL2 and TGF-β1/TGF-β2 (Supplementary Figure S9), suggesting a broader regulatory network that links this ceRNA axis to immune response and epithelial-mesenchymal transition pathways.

iii. Gene set enrichment analysis

Gene ontology (GO) and Kyoto Encyclopedia of Genes and Genomes (KEGG) pathway analyses for lnc-HNRNPUL2, *SFTPD* mRNA, and miR-335-5p were performed using the Enrichr tool (https://maayanlab.cloud/Enrichr/). Results confirmed significant enrichment in immune-related processes, molecular functions related to RNA binding and immune receptor activity, and cellular components, including extracellular exosomes and membrane (Supplementary Figure S10). These findings support the biological relevance of the proposed axis in CRC pathogenesis.

**Fig. 2:**
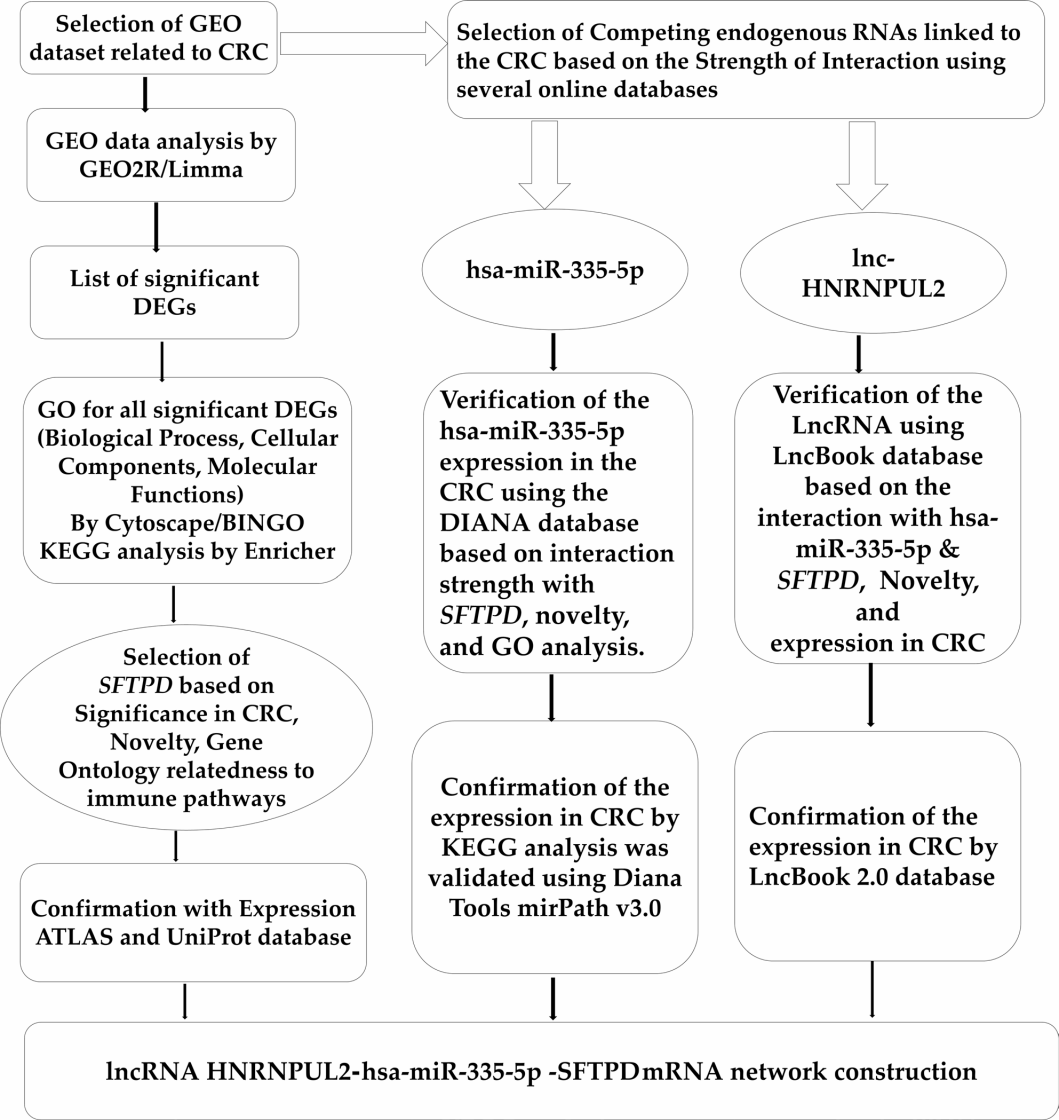
Schematic figure showing the summary of the Bioinformatics setups in the study

We adopted an in silico approach integrated with previous literature evidence [30, 31] to verify the chosen RNA panel. Moreover, it was assessed in silico that there is a direct interaction between lnc-HNRNPUL2 and TGF-β1 and TGF-β2, as shown in the ensemble database (https://www.ensembl.org/index.html) (Supplementary Figure S9). The selection criteria and bioinformatics validation for each component of the *SFTPD*/miR-335-5p/lnc-HNRNPUL2 axis are summarized in Table S3 & Fig. 2.

### Extraction of RNAs from serum and HT29 cells

Total RNA was extracted from the serum and the HT29 cells using the miRNeasy RNA isolation kit (Qiagen, Hilden, Germany), according to the manufacturer’s instructions. The RNA samples were dissolved in 35 µL RNase-free water, and the concentration and purity were determined by a NanoDrop spectrophotometer (Thermo Scientific, USA). The miScript II RT Kit (Qiagen, Hilden, Germany) was used to produce total cDNA, and the iScript kit (BioRad, USA) was used to generate cDNA for mRNA.

### Quantification of the RNA network panel by real-time PCR

The QuantiTect SYBR Green PCR Kit and RT^2^ SYBR Green Mastermix were used to measure the expression of *SFTPD* mRNA (NM_003019) in serum samples and HT29 cells. The GAPDH Primer Assay (NM_001101) was used as a housekeeping control (Supplementary Table S4). Relative expression levels for lnc-HNRPUL2 were analyzed by HS_HNRNPUL2_699339 QuantiNova LNA Probe PCR Assay (ENST00000540127.1, Qiagen, Helman, Germany).

miRNA expression levels (MIMAT0000765) were quantified using the miScript SYBR Green PCR Kit (Qiagen, Hilden, Germany), and the resulting signals were normalized to the reference gene RNU6-2 (SBH0614997). Each reaction was performed thrice. We used the Livak method, RQ = 2^-ΔΔCt^, for quantifying RNA expression.

We used the Rotor-Gene Q (Qiagen, Hilden, Germany) to calculate the threshold cycle (Ct) for each sample. A Ct value of more than 36 was considered a negative result; the melting curves.

### Estimation of TGF-β1 by enzyme-linked immunosorbent assay (ELISA)

TGF-β1 levels in the sera samples and cells were measured using the TGF beta-1 ELISA Kit with Plates (Invitrogen, Carlsbad, CA, USA), according to the manufacturer’s instructions. The ELISA results were normalized by total protein concentrations measured using the BioRad Protein Assay reagent. The total protein in serum was measured using the BioRad Bradford protein assay (Biorad, CA, USA).

### Cell culture and viability assay

The human colorectal cancer cell line HT29 was selected as a representative model of colorectal adenocarcinoma for initial functional validation of the *SFTPD* axis. The human colorectal cancer cell line HT29 was purchased from Nawah Scientific Research Institute (Egypt). Cells were maintained in RPMI-1640 medium supplemented with 2 mM L-glutamine and 10% fetal bovine serum (Biowest, USA). Cells were incubated in a humidified atmosphere (37 °C, 5% CO_2_). Cellular viability was determined by the MTT assay. For that purpose, both control Small interfering RNA (siRNA) and targeted siRNA-transfected clones were cultured in 96-well plates (5,000 cells/well) in a final volume of 100 µL to a confluence of 50%. HT29 cells were treated with 20 nM negative control siRNA (AllStars Negative; Qiagen).

We used the lowest possible siRNA concentration to achieve a therapeutic effect to exclude any consequences of the cytotoxicity of the used siRNA, by carrying out a siRNA dose-response curve to choose the optimal siRNA concentration. The culture media was replaced 72 h later by the MTT reagent, which was incubated for 4 h at 37 ℃. Finally, equal volumes of dimethyl sulfoxide were added to each well to dissolve the crystals, and the absorbance was measured at 570 nm, the reference wavelength being 690 nm.

### siRNA transfection

siRNA against lnc-RNA, lnc-HNRNPUL2 (FlexiTube gene solution siRNA; GS400654), and control siRNA were purchased from Qiagen (Germany). For transfection, HT29 cells were cultured to 50% confluence overnight, and siRNA (20 nM) was transfected using Lipofectamine 3000 (Invitrogen), according to the manufacturer’s recommendations. Two days later, cells were collected by trypsinization, washed twice with phosphate-buffered saline, and subjected to RNA extraction.

### Statistical analysis

The heatmap and box plots representing the relative fold gene expression of serum biomarkers were drawn using the (https://www.bioinformatics.com.cn/en) web tool. The chi-squared test was used to analyze the categorical demographic data, which was presented as frequencies and percentages. The Shapiro-Wilk test was used to determine the normality of the distribution of numeric variables. The normally distributed data were expressed as Mean ± SD and analyzed using the unpaired samples T-test to quantify the significant differences between the two groups or by the analysis of variance post hoc Tukey test to quantify the significant differences between the 3 groups. The non-normally distributed data were represented by their medians and interquartile range (25–75%) and analyzed using Kruskal–Wallis Dunn’s multiple comparison post hoc test. The expression correlation between serum biomarkers was assessed using Spearman’s Rho coefficient test between the control and CRC groups. Receiver operating characteristic (ROC) analysis was performed to evaluate the diagnostic performance of individual and combined biomarkers. For each biomarker, the continuous expression value (fold-change for RNAs, concentration for proteins) was used as the test variable to classify samples as CRC or non-malignant (combining benign and healthy controls). The ROC curve was plotted by calculating the sensitivity and 1 specificity at all possible cut-off points. The optimal cut-off was determined as the point maximizing Youden’s Index (J = sensitivity + specificity − 1). The area under the curve (AUC) with its 95% confidence interval was computed using the DeLong method [22]. Combined biomarkers were calculated by taking the average of the fold change of *SFTPD*/miR-335-5p/ lnc-HNRNPUL2, and then the ROC curve was plotted. While groups were frequency-matched for age and sex, BMI differed significantly. All biomarker comparisons employ statistical adjustment for these variables where appropriate. Statistical analyses were performed using the IBM SPSS Statistics 26 software. A *P*-value < 0.05 was considered statistically significant.

## Results

### Description of population characteristics

Significant intergroup differences were observed for BMI (*p* = 0.006), with patients in the malignant cohort exhibiting the highest values. Colonoscopic findings differed markedly between groups (*P* < 0.001): control subjects demonstrated no detectable lesions, benign cases were predominantly characterized by polyps, and malignant cases most frequently presented with masses. Histopathological profiles also varied significantly (*P* < 0.001), with adenocarcinoma comprising the majority of malignant tumors. Within the malignant group, grade 2 and stage II disease were most prevalent, and metastasis and relapse were documented in 17.3% and 35.8% of patients, respectively. In contrast, age, sex, smoking status, and family history did not differ significantly among the study groups (Table 1).

### Expression of the serum SFTPD panel among the studied groups

**Fig. 3:**
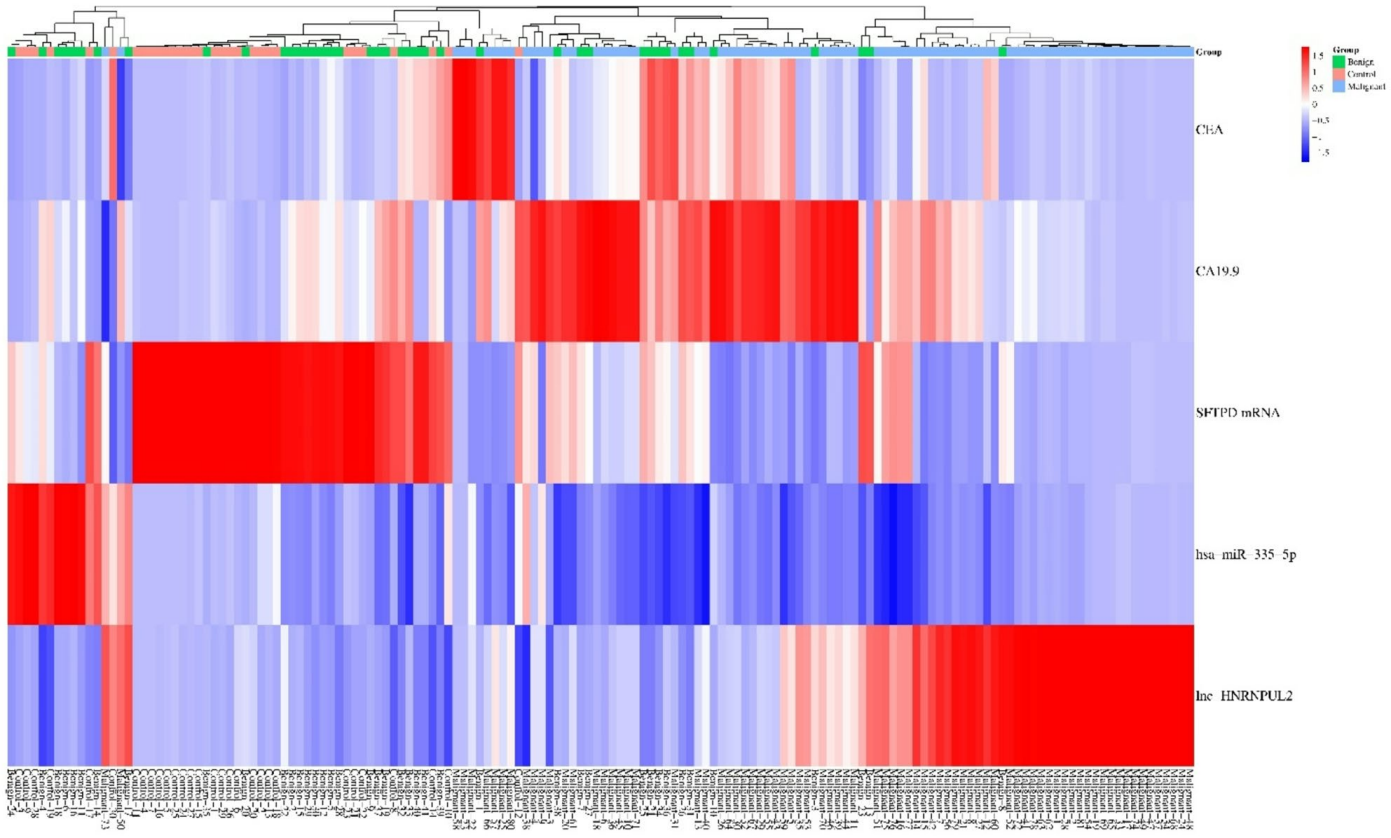
Heatmap analysis revealed the expression pattern of lnc-HNRNPUL2, *SFTPD* mRNA, hsa-miR-335-5p, CEA, and CA19.9 among the control, benign, and malignant group samples

**Fig. 4:**
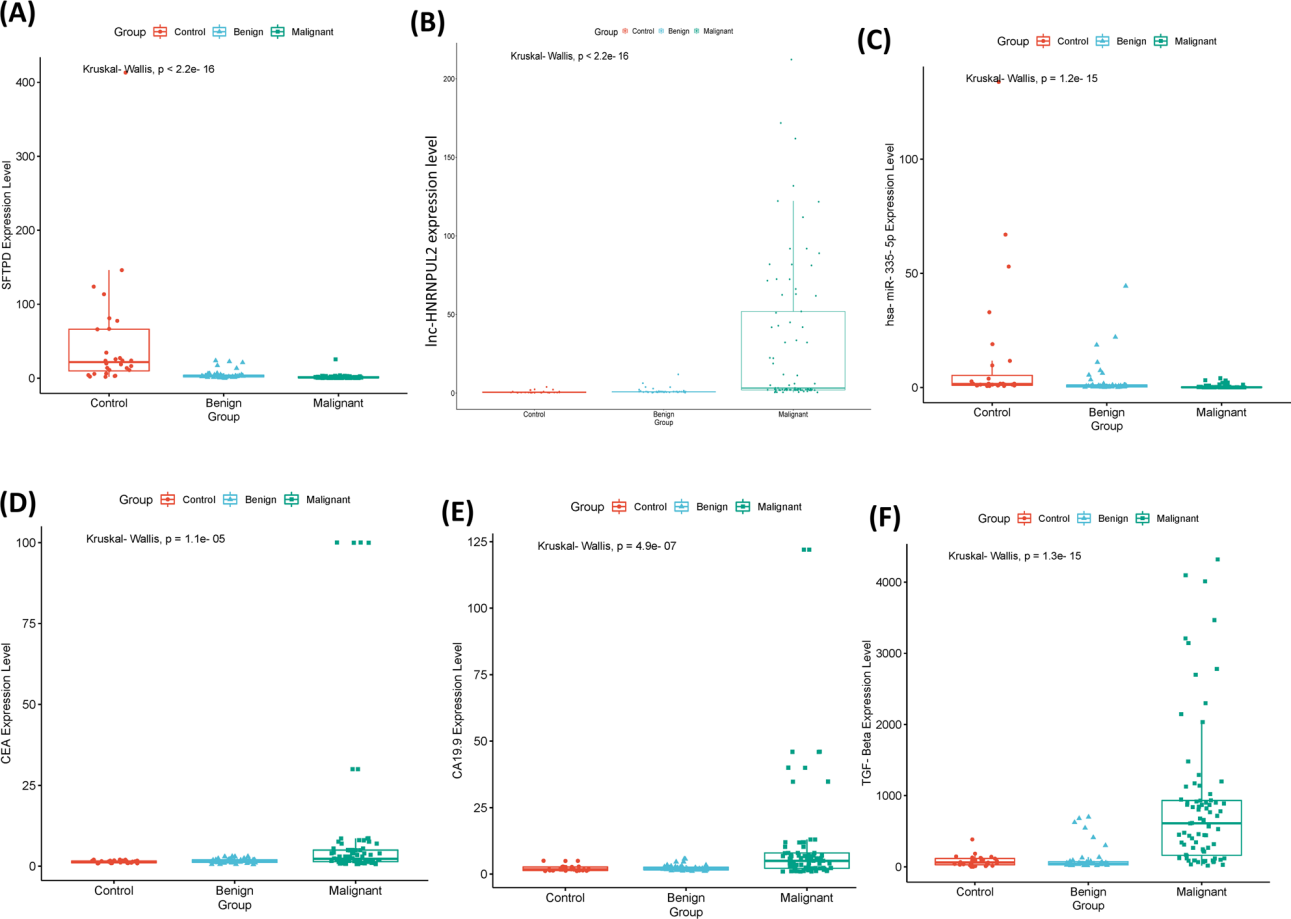
Box plots represent the difference in expression levels of serum biomarkers. *P*-value < 0.05 represents a significant difference among groups. **A** SFTPD mRNA expression levels, (**B**) lnc-HNRNPUL2 expression levels, (**C**) hsa-miR-335-5p expression levels, (**D**) CEA expression levels, (**E**) CA19-9 expression levels, and (**F**) TGF-β1 expression levels

The heatmap of Fig. 3 clearly shows the inverse expression patterns of lnc-HNRNPUL2 with both *SFTPD* mRNA and hsa-miR-335-5p, as lnc-HNRNPUL2 was highly expressed in the malignant group, while *SFTPD* mRNA and hsa-miR-335-5p were highly expressed in the control and benign groups, with a remarkable increase in expression of both CEA and CA19.9 in the malignant group (Figs. 3 and 4).

The expression of lnc-HNRNPUL2 was significantly elevated by 3.4-fold in the CRC group and 1.7-fold in the benign group compared with the control group. TGF-β levels similarly increased 5.34-fold in the CRC group and 2.79-fold in the benign group. In contrast, the *SFTPD* mRNA and hsa-miR-335-5p levels decreased significantly by 2.5-fold and 2.46-fold in the CRC group, and 1.4-fold and 1.27-fold in the benign group, respectively. CA19.9 levels increased significantly by 1.72-fold in the CRC group but only by one-fold in the benign group. CEA showed a slight increase of 1.76- and 1.18-fold in the CRC and benign groups, respectively (Table 2). For descriptive statistics and initial group comparisons (e.g., Table 2; Fig. 4), values represent unadjusted expression levels. Statistical adjustments for potential confounders (age, sex, BMI, and tumor stage) are specifically incorporated into multivariate regression models where appropriate, as detailed in the Methods section.

**Table 2:** Expression levels of lnc-HNRNPUL2, *SFTPD* mRNA, hsa-miR-335-5p, TGF-β, CEA, and CA19.9 in control, benign, and malignant groups

Group	Control	Benign	Malignant	*P*-Value
lnc-HNRNPUL2 Mean Rank	0.41 (0.23–3.39) 30.73	0.89 (0.31–11.92)^a^ 53.79	3.24 (0.5-212.22)^a, b^ 104.94	1.74E-17
*SFTPD* Mean Rank	21.7 (1.13-413.14) 128.47	2.79 (0.03–22.36)^a^ 89.29	0.96 (0.01–25.36)^a, b^ 50.78	1.45E-16
miR-335-5p Mean Rank	1.46 (0.67-133.88) 122.43	0.63 (0.06–44.44)^a^ 95.78	0.05 (0.01–4.04)^a, b^ 49.73	4.68E-16
TGF-β Mean Rank	11.5 (2–77) 20.13	44 (17–700)^a^ 56.29	610 (16-4320)^a, b^ 107.6	5.7615E-16
CEA Mean Rank	1.4 (0.9-2) 52.45	1.5 (0.6-3) 62.26	2.4 (0.7–100)^a, b^ 92.62	6.00E-06
CA19.9 Mean Rank	1.75 (1.2-5) 54.85	1.9 (1.1–5.7) 56.33	5 (1-122)^a, b^ 94.73	3.39E-07

Data were expressed as the median and interquartile range (25–75%). The statistically significant difference between the 3 groups was analyzed using the Kruskal-Wallis analysis of variance test

The *P*-value < 0.05 was considered statistically significant. ^a^*P*< 0.05 compared with the control group, and ^b^*P*<0.05 compared with the benign group

Kruskal-Wallis analysis of variance test revealed that the levels of lnc-HNRNPUL2 and TGF-β were significantly upregulated in the CRC group compared with the healthy control and benign groups (*P-*value < 0.05), while those of *SFTPD* mRNA and miR-335-5p were significantly downregulated in the CRC group and benign group compared with the healthy control group (Fig. 4; Table 2).

**Fig. 5:**
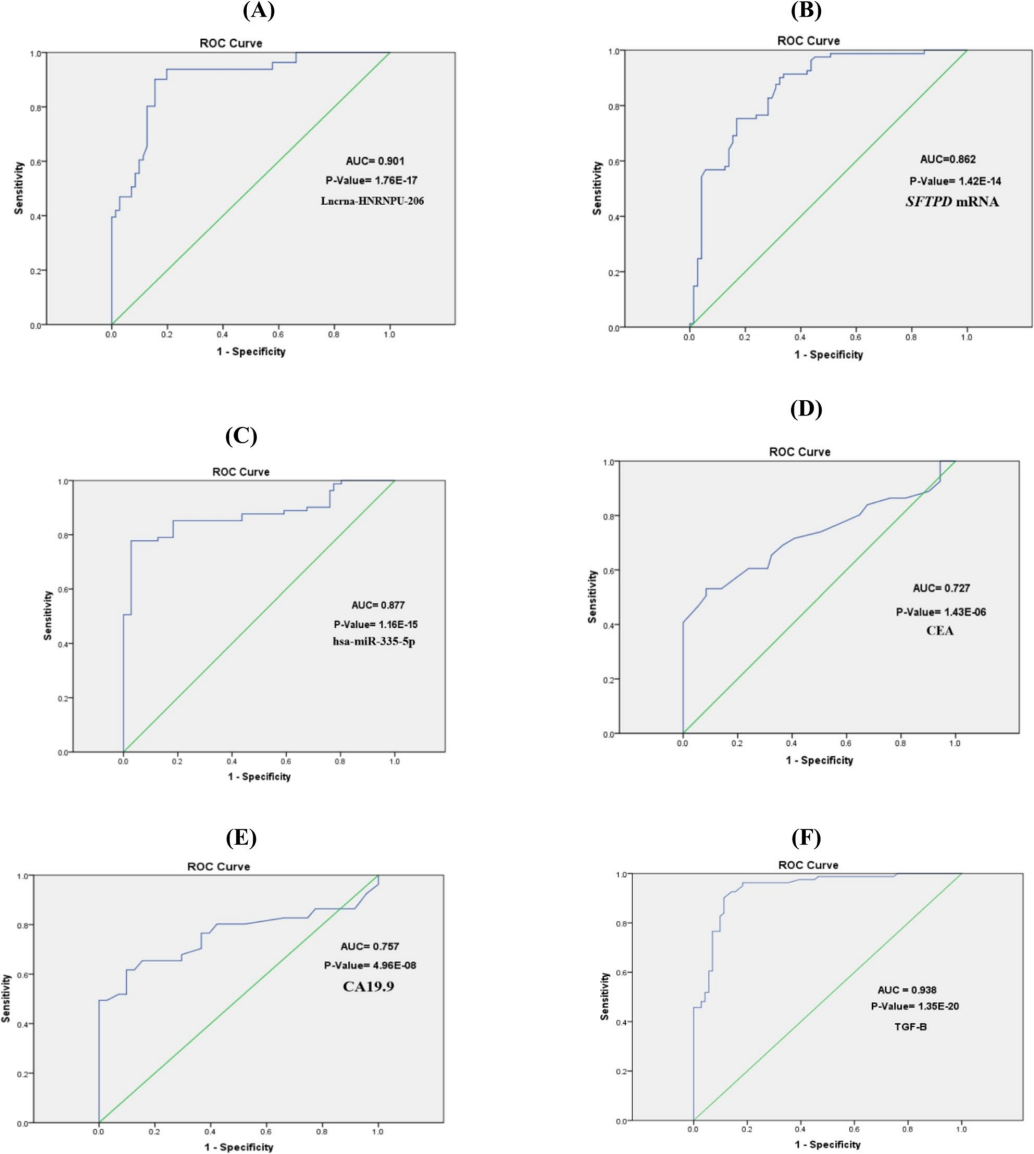
ROC curve analysis for (**A**) lnc-HNRNPUL2, (**B**) *SFTPD* mRNA, (**C**) hsa-miR-335-5p, (**D**) CEA, (**E**) CA19.9, and (**F**) TGF-β, correlated with CRC group against non-malignant patients

ROC curves were used to estimate the diagnostic power of the selected *SFTPD* panel in comparison to the traditional biomarkers CEA and CA19.9. The optimal cut-off values of lnc-HNRNPUL2, *SFTPD* mRNA, hsa-miR-335-5p, TGF-β, CEA, and CA19.9 in CRC patients compared with the non-malignant group were 1.63, 2.5, 0.19, 16.5, 2.05, and 2.35, respectively. lnc-HNRNPUL2, *SFTPD* mRNA, hsa-miR-335-5p, and TGF-β had high sensitivity values of 86.9%, 77.9%, 96.9%, and 98.8%, while CEA and CA19.9 had low sensitivity values of 54.3% and 69.5%, respectively. Preliminary analysis indicated that the *SFTPD* biomarker panel combined showed a high sensitivity (Table 3; Fig. 5). To assess the combined diagnostic utility of the identified biomarkers, we constructed a multivariate logistic regression model using lnc-HNRNPUL2, SFTPD mRNA, and hsa-miR-335-5p as continuous predictors. Consistent with our methodology, this model inherently accounts for potential confounders such as age, sex, BMI, and tumor stage, as specified in the Statistical Analysis section. This model demonstrated strong discriminatory power with an apparent Area Under the Curve (AUC) of 0.936. To ensure the generalizability and minimize overfitting, we performed rigorous internal validation using 10-fold cross-validation. This yielded a robust cross-validated AUC of 0.902, with a sensitivity of 85.2% and specificity of 83.1%. The minimal difference between the apparent and cross-validated AUCs (approximately 3.6%) suggests a low risk of overfitting, confirming the model’s reliable performance on new data. Further validation with 1,000 bootstrap resamples yielded an optimism-corrected AUC of 0.895, and the Hosmer-Lemeshow test (*P* = 0.598) indicated a good model fit.The small difference between apparent and validated AUCs (~ 4%) suggests minimal overfitting. The cross-validated AUC of 0.902 represents a more realistic estimate of performance on new data.

**Table 3:** Optimal cut-off levels, sensitivity, specificity, positive predictive value (PPV), negative predictive value (NPV), and area under the curve of investigated serum biomarkers by receiver operating characteristic (ROC) curve analysis correlated with the CRC group against non-malignant patients

Biomarker	Area under the curve	Cut-off	sensitivity	Specificity	PPV	NPV
lnc-HNRNPUL2	0.901	1.63	86.9%	84.5%	90.1%	84.5%
*SFTPD* Mrna	0.862	2.5	77.9%	78.8%	82.7%	73.2%
hsa-miR-335-5p	0.877	0.19	96.9%	79.3%	77.8%	97.2%
TGFβ	0.938	16.5	98.8%	25.4%	60.2%	94.7%
CEA	0.727	2.05	54.3%	87.3%	83%	62.6%
CA19.9	0.757	2.35	69.5%	65.7%	70.4%	64.8%
Combined(*SFTPD*/miR-335-5p/lnc-HNRNPUL2)	0.901	1.5	100%	62%	75%	100%
Multivariate Panel (Logistic Model)†	0.936	0.642	88.9%	85.9%	88.9%	85.9%
10-Fold Cross-Validated Panel‡	0.902	0.642	85.2%	83.1%	85.2%	83.1%

† The “Multivariate Panel” represents the predicted probability from a logistic regression model incorporating *SFTPD*/miR-335-5p/lnc-HNRNPUL2 as continuous predictors. ‡ The “10-Fold Cross-Validated Panel” represents performance metrics derived from 10-fold cross-validation of the multivariate logistic regression model, providing a more realistic estimate of generalizability

We performed a binary logistic regression with lnc-HNRNPUL2, *SFTPD* mRNA, and hsa-miR-335-5p as continuous predictors. All three were highly significant independent predictors (*P* < 0.001 for each) (Table 4). Cox regression analysis highlighted the prognostic utility of the model, with a significance value of *P* < 0.05 (Supplementary Table S4).

**Table 4:** Multivariate Logistic Regression Analysis for CRC Diagnosis (CRC vs. Non-malignant)

Predictor	β Coefficient	Standard Error (SE)	Wald Statistic	*P*-value	Odds Ratio (OR)	95% CI for OR
lnc-HNRNPUL2	0.034	0.006	35.14	< 0.001	1.035	1.023–1.047
*SFTPD* mRNA	-0.176	0.031	32.06	< 0.001	0.839	0.790–0.891
miR-335-5p	-2.614	0.528	24.52	< 0.001	0.073	0.026–0.206
Constant	2.378	0.560	18.04	< 0.001	10.786	

Model Summary Statistics: Number of observations: 152 (81 CRC vs. 71 Non-malignant), Model χ² (df = 3): 85.67, *P* < 0.001,Nagelkerke R²: 0.58, Hosmer-Lemeshow test: χ² = 6.45, *P* = 0.598 (good fit), Apparent AUC (Training): 0.936, 10-Fold Cross-Validated AUC: 0.902

### Correlation between the expression levels of the investigated parameters among the study groups

Importantly, we found a strong negative correlation of lnc-HNRNPUL2 with both *SFTPD* mRNA and hsa-miR-335-5p, while it is positively correlated with TGF-β, CA19.9, and CEA. A significant positive correlation was observed between *SFTPD* mRNA and miR-335-5p. This co-downregulation pattern suggests they may be coregulated in the context of CRC, though it does not fit a simple sponge model where lnc-RNA upregulation leads to miRNA sequestration and subsequent target mRNA derepression. TGF-β positively correlated with lnc-HNRNPUL2, CA19.9, and CEA and negatively correlated with *SFTPD* mRNA and miR-335-5p (Supplementary Table S4).

### Exploratory analysis of biomarker association with metastasis and early outcomes

Given the mean follow-up duration of 14 ± 6.9 months—insufficient for robust overall survival analysis in CRC—these findings should be interpreted as hypothesis-generating rather than prognostic validation. miR-335-5p: Significantly lower in metastasized vs. non-metastasized (0.04 vs. 0.44, *P* = 3.03E-04) TGF-β: Lower in metastasized vs. non-metastasized (524.8 vs. 942.4, *P* = 0.02).

lnc-HNRNPUL2: Higher in metastasized (49 vs. 28.4, *P* = 0.265) (Supplementary Table S5& S6). Kaplan-Meier analysis with log-rank testing revealed no statistically significant association between biomarker expression levels and survival outcomes during the available follow-up period (*P* > 0.05, Supplementary Figure S11). Given the short follow-up period, we focused the Cox regression analysis on metastasis as an endpoint. Low hsa-miR-335-5p expression emerged as a significant predictor of metastasis (HR = 3.85, 95% CI 1.42–10.45, *P* = 0.008). This significance was maintained in a multivariate Cox regression analysis that inherently adjusted for key confounders including age, BMI, and tumor stage (HR = 3.12, 95% CI 1.08–9.01, *P* = 0.035). TGF-β showed a trend toward significance (*P* = 0.075), while lnc-HNRNPUL2 and *SFTPD* mRNA were not significant predictors in this analysis (Supplementary Table S7, Supplementary Figure S11).

### Validation of the SFTPD panel in vitro

**Fig. 6:**
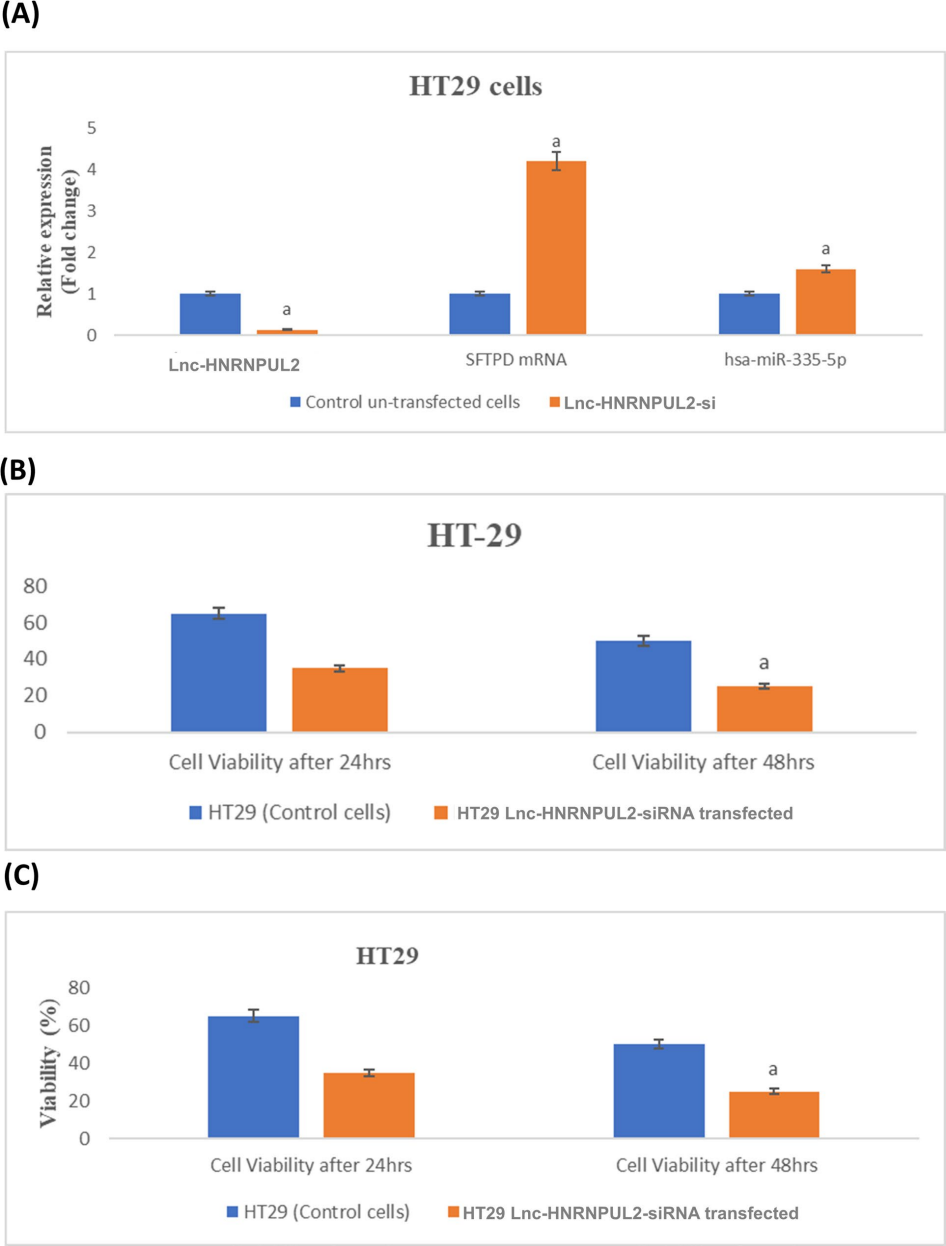
**A** Quantitative polymerase chain reaction data for the expression levels of lnc- HNRNPUL2, *SFTPD* mRNA, and hsa-miR-335-5p in the control un-transfected and the lnc-HNRNPUL2-si-transfected HT29 cells. **B** The cell count (in millions) after 24 and 48 h of cell transfection relative to the un-transfected control cells. **C** The cell viability of the transfected cells compared with control un-transfected cells after 24 and 48 h. While (a) represents a statistically significant difference (*P*-value < 0.05) between the control and transfected cells, determined by an independent sample T-test

To provide preliminary functional validation of the axis, we transfected HT29 cells with siRNA targeting lnc-HNRNPUL2. Silencing the expression of the lnc-RNA led to a significant increase in the levels of miR-335-5p and *SFTPD* mRNA, which was consistent with the in silico data analysis and serum data, supposing the ability of lnc-HNRNPUL2 to inhibit miR-335-5p, which in turn regulates *SFTPD* mRNA expression (Fig. 6A). Interestingly, lnc-HNRNPUL2 knockdown negatively affected cell viability after 24 h. Still, the effect was not appreciable after 72 h (Fig. 6B, C), suggesting that the cells may have adopted alternative mechanisms to overcome lnc-HNRNPUL2 knockdown. These data are consistent with the proposed regulatory network and suggest that lnc-HNRNPUL2 may contribute to CRC cell growth. Finally we can summarize the following findings to present the supporting evidence for the *SFTPD*/miR-335-5p/lnc-HNRNPUL2 axis across three levels: (1) Bioinformatic prediction of high-confidence interactions from multiple databases, (2) Clinical correlation showing a significant inverse relationship between lnc-HNRNPUL2 and miR-335-5p in patient sera, and (3) Functional perturbation, where siRNA-mediated knockdown of lnc-HNRNPUL2 in HT29 cells resulted in the expected reciprocal modulation of miR-335-5p and *SFTPD* expression. While these layers of evidence collectively support the biological plausibility of the axis, they do not establish direct molecular binding, which would require dedicated interaction assays such as luciferase reporters or RNA pull-downs.

## Discussion

CRC develops from multiple genetic and epigenetic changes in precursor lesions (adenomas and serrated lesions) that progress to become adenocarcinomas. The prevention of CRC requires an early diagnosis of the disease during the screening of average-risk, asymptomatic individuals [32]. Colonoscopy, the gold standard for CRC screening, is invasive, costly, has low compliance rates, prerequisite patients’ specific preparations, and can be complicated by bleeding or perforation, moreover, this method has limited applicability in long-term monitoring [33]. These drawbacks emphasize the critical need for alternative, minimally invasive biomarkers or therapeutic targets for CRC [32].

Recently, liquid biopsy has been identified as a significant approach for CRC diagnosis, prognosis, and progression monitoring that can effectively overcome the obstacles of tissue biopsies [34]. Liquid biopsy is the analysis of tumor cells using biomarkers found in body fluids such as blood, urine, ascites, and cerebrospinal fluid [33]. Liquid biopsy offers several advantages over tissue biopsy, including ease of sampling, highly sensitive early diagnosis, non-invasive cancer screening, patient categorization, and surveillance when surgery is not optional or when insufficient tissue samples are available [35]. Furthermore, it was proven to detect genetic and epigenetic tumor microenvironments and track genomic evolution, in addition to the acquired resistance [36, 37]. Blood biomarkers had previously proven to be a non-invasive method for CRC screening, especially in its early stages (stage I or premalignant stage) [38].

Although CEA is the most widely accepted blood-based biomarker for CRC, it is also a biomarker for a variety of other malignancies, making it insufficient alone for CRC diagnosis. As a result, CA19.9, cancer antigen 125 (CA125), cancer antigen 72 − 4 (CA72-4), and tumor-associated glycoprotein 72 (TAG 72) have been recognized and suggested for CRC diagnosis [39].

The immune microenvironment is a specialized milieu with the ability to modify cancer biology and is linked to cancer prognosis and therapeutic responses [14]. The interaction between cancer cells and the immune system is complex. Tumor cells and the immune microenvironment produce immunosuppressive cytokines, such as interleukin-10, prostaglandin E2, TGF-β, and vascular endothelial growth factor (VEGF), which are implicated in cancer proliferation, growth, and invasion. These cytokines confer an immunosuppressive state upon the tumor cells, enabling them to evade immune surveillance [40].

The potential cross-talk between serum miRNA, lnc-RNA, and mRNA has been thoroughly studied, and RNA regulatory axis analysis has emerged as a useful tool for identifying potential prognostic biomarkers in various cancers [41]. we investigated the *SFTPD* biomarker panel (*SFTPD* mRNA, lnc-HNRNPUL2, miR-335-5p, and TGF-β) in CRC patients in comparison to benign colorectal neoplasms and healthy controls.

Our in silico analysis highlighted a potential molecular network in CRC involving *SFTPD* mRNA, miR-335-5p, and lnc-HNRNPUL2. To the best of our knowledge, this RNA panel is reported here for the first time in the context of CRC. To validate this panel, we studied the expression of the biomarkers in sera from CRC patients, patients with chronically benign colorectal lesions, and healthy volunteers of different ages and genders. We found that the expression of miR-335-5p and *SFTDP* mRNA was significantly depleted in the sera of CRC patients compared to controls. These depleted levels were associated with high expression of lnc-HNRNPUL2 in the serum, which had lower expression levels in the control group. These data suggest that the *SFTDP*/miR-335-5p/lnc-HNRNPUL2 axis is deregulated specifically in CRC. The axis was validated to be present in the tested CRC cell line, indicating that it could be a key player in CRC pathogenesis.

The diagnostic performance metrics, particularly the high sensitivity observed for the combined panel, were derived from a single cohort and are therefore subject to overfitting.

The *SFTPD* gene is located at position 10q22.2-23.1 in the human genome [42]. The *SFTPD* protein, an innate immune molecule, influences the production of various cytokines and chemokines, such as TNF-α and TGF-β, during infection, allergy, and inflammation [21]. *SFTPD* has also been implicated as an immune surveillance molecule against tumors [10]. Its interaction with a variety of cancer cells (leukemia, lung, prostate, and pancreatic) has been shown to inhibit cancer progression, migration, and invasion while accelerating apoptosis [43]. In our study, *SFTPD* mRNA was significantly downregulated while the expression of TGF-β1 was significantly upregulated in the CRC group compared with the benign and control groups.

Mangogna et al. found *SFTPD* expression to be lower in lung, gastric, and breast cancers and higher in ovarian cancer compared with normal tissues. They also reported that the presence of *SFTPD* in the lungs could be correlated with a better prognosis, while its presence in non-pulmonary malignancies might be associated with a poor prognosis [10].

In ovarian cancer, high *SFTPD* expression is associated with poor prognosis, while low *SFTPD* expression significantly improved overall survival and progression-free survival compared to higher *SFTPD* expression [10]. On the other hand, in our study, low *SFTPD* expression is associated with poor prognosis. This contradiction indicates a higher level of complexity in the role of *SFTPD* in cancer. It is likely that *SFTPD* behaves differently in different tissues and cell types, according to the composition of the tumor microenvironment [10]. This agrees with Pandit et al., who clarify that changes in serum levels of *SFTPD* protein, in pathophysiological conditions, directly and differently affect the behavior of systemic immune cells and thus determine the disease outcome [44].

TGF-β1 upregulation in the tumor microenvironment impairs immune surveillance, allowing tumors to escape, migrate, and develop more resistance to anti-tumor immune responses [21]. Kaur et al. found that in pancreatic cancer cell lines, human *SFTPD* suppressed TGF-β-induced epithelial-to-mesenchymal transition (EMT) by inhibiting Smad phosphorylation. They also reported that the recombinant fragment of human *SFTPD* stimulated apoptosis in these cells through the TNF-α/Fas pathway, regardless of the p53 status [21].

Given that Surfactant protein D (SP-D)—encoded by the *SFTPD* gene—participates in immune defense, lipid metabolism, and cardiometabolic disease, its genetic modulation provides a biologically plausible link to the mechanisms addressed in our findings [45]. SP-D aids in the neutralization, agglutination, and clearance of viruses, as well as apoptotic cells, and the reduction of the inflammatory response to infection. *SFTPD* acts as a tumor suppressor by: і. Stimulating TNF-α and IFN-γ production [46]. ⅱ. Retaining T cells is hyporesponsive to promote CTLA4 expression, decrease T cell proliferation, reduce allergen-induced Th2 cytokine production, and control apoptosis [46]. ⅱi. SP-D antagonizes epidermal growth factor receptor (EGFR) and suppresses the downstream EGFR signaling process, which accordingly inhibits the metastases of cancer cells [47]. іv. SP-D regulates the lipopolysaccharides (LPS)-induced apoptosis by interacting with the Toll-Like Receptor 4 (TLR4) and CD14 and activating the P38 MAPK signaling pathway [48].

This is consistent with the findings of Mahajan et al., who demonstrated that SP-D and a recombinant fragment of human SP-D triggered G2/M phase cell cycle arrest as well as dose and time-dependent apoptosis in the eosinophilic leukemia cell line (AML14.3D10). Following rfhSP-D treatment, several apoptotic indicators, such as activated p53, cleaved caspase 9, PARP, and G2/M checkpoints, were significantly boosted, along with lowered levels of survival factors such as HMGA1 [49].

In colorectal cells, *SFTPD* regulates adaptive and innate immune responses against commensal bacteria in the colon mucosal surfaces [50]. The rs2243639 polymorphism in *SFTPD* (Ala160Thr) has been shown to affect the molecule’s oligomeric state, protein function, and protein level in the blood [51]. Tanaka et al. discovered that the rs2243639 polymorphism is strongly associated with ulcerative colitis susceptibility in Japanese [52].

Lnc-RNA expression is linked to the occurrence and progression of many diseases, including cancers [53]. Although some lnc-RNAs have been implicated in the dysregulation of proliferation, apoptosis, migration, invasion, and chemoresistance in CRC [26], the involvement of several other lnc-RNAs is yet unexplored [54]. Zhang et al. found that lnc-RNA H19 expression was significantly increased in primary and metastatic CRC and was associated with a poor prognosis. Abnormal H19 expression enhanced the metastasis of CRC cells in vitro and in vivo and triggered EMT [54]. Lnc-HNRNPUL2 was selected based on bioinformatics predictions indicating potential binding sites for miR-335-5p (Supplementary Figure S6) and upregulation in CRC tissues (Supplementary Figure S7). It has also been linked to colon cancer growth and metastasis through activating Ras/MAPK signaling pathways [55]. Our data suggest that lnc-HNRNPUL2 may act as a regulatory axis of RNA, potentially sequestering miR-335-5p and thereby modulating the expression of its target genes, including *SFTPD*.

miR-335-5p is abnormally expressed in many malignancies, and its expression varies with cancer type [56–58]. For instance, miR-335-5p was significantly downregulated in non-small cell lung cancer (NSCLC) [30], breast cancer [59], renal cell carcinoma [60], and gastric cancer [61]. In our study, miR-335-5p was significantly downregulated in the CRC group compared with the benign and control groups. miR-335-5p has been shown to suppress cell proliferation, migration, and invasion in CRC by downregulating lactate dehydrogenase B [62]; miR-335-5p has been shown to target TGF-β1 and suppress TGF-β-induced EMT in non-small cell lung cancer [30], and to inhibit invasion and metastasis in CRC by targeting lactate dehydrogenase B [60]. Our bioinformatics analysis further supports miR-335-5p as a regulator of both *SFTPD* and TGF-β (Supplementary Figures S3 & S8), positioning it as a central node in the proposed network. inhibit invasion and metastasis of thyroid cancer cells by targeting intercellular adhesion molecule-1 [63], and suppress TGF-β1-induced EMT in NSCLC via Rho-associated protein kinase 1 [30]. Also, Gao et al. found that miR-335-5p functioned as a tumor suppressor in gastric cancer, and its overexpression reduced proliferation, invasion, and metastasis while inducing apoptosis in vitro. Furthermore, miR-335-5p promoted cell cycle arrest by downregulating the cell cycle-associated proteins–cyclin-dependent kinase 6 (CDK6), CDK4, and cyclin D1–in the G_0_ and G_1_ phases [64].

We observed a strong negative correlation between lnc-HNRNPUL2 and miR-335-5p and a positive correlation between miR-335-5p and *SFTPD* mRNA across study groups. These correlations are consistent with the hypothesis that lnc-RNA can act as a “proposed regulatory axis ” for miRNAs by competitively binding to them, thereby influencing the miRNA-mediated regulation of downstream target genes [13]. Moreover, a significant positive correlation was observed between *SFTPD* mRNA and hsa-miR-335-5p, which signifies that the miRNA could regulate the target mRNA. Moreover, Lu et al. investigated potential type 2 diabetes mellitus (T2DM) biomarkers and discovered that the *SFTPD* expression was reduced in obesity and impaired glucose tolerance, which are directly linked to T2DM development. They additionally verified that *SFTPD* was regulated by hsa-miR-335-5p [65]. The miR-335 upregulation acted as a tumor suppressor by inhibiting Bcl-w, an antiapoptotic member of the Bcl-2 family, which has been found to be closely associated with cancer formation and progression, in NSCLC [66].

Mostly, miRNAs bind to the 3′ UTR of target mRNAs to degrade them and repress translation. However, miRNAs have also been shown to interact with other regions, including the 5′ UTR, coding sequence, and promoters, to stimulate translation or regulate transcription [67]. Vasudevan et al. reported that, under specific circumstances, AU-rich elements in TNF/TNF-α mRNA function as signals for translation activation, and this process involves miRNAs [68].

Moreover, available evidence suggests that microRNAs play a role in TGF-β1 signaling-induced EMT [69]. MiR-335 targets and consequently suppresses genes in TGF-β non-canonical pathways, including the Rho-associated coiled-coil containing protein (ROCK1) and MAPK1, resulting in decreased phosphorylation of downstream pathway members [31]. MiR-335 has also been shown to suppress gastric cancer cell invasion and metastasis by directly targeting the mRNA transcripts of specificity protein 1 (SP1) and Bcl-w (BCL2L2), which are part of the PI3K/AKT pathway involved in TGF-β non-canonical signaling [30, 68]. Based on the mentioned evidence, we suggest a possible regulatory mechanism for the TGF-β by miR-335 in the CRC. Although direct binding interactions were not experimentally validated here, our functional siRNA knockdown of lnc-HNRNPUL2 in HT29 cells resulted in the expected reciprocal changes: increased miR-335-5p and *SFTPD* expression, alongside decreased cell viability. This functional perturbation supports the plausibility of the proposed regulatory axis. Our in vitro siRNA knockdown of lnc-HNRNPUL2 in HT29 cells provided confirmatory evidence that this lnc-RNA is functionally associated with miR-335-5p and *SFTPD* change in expression in a CRC context. While more comprehensive functional studies—including migration, invasion, and apoptosis assays in multiple cell lines—are needed to fully elucidate the mechanistic role of this axis, our data support its biological relevance and diagnostic potential.

Interestingly, we observed a positive correlation between miR-335-5p and *SFTPD* mRNA levels in patient sera, with both being downregulated in CRC. This was contrary to the initial ceRNA model prediction, in which lnc-HNRNPUL2 upregulation would sponge miR-335-5p and lead to *SFTPD* derepression. This discrepancy highlights the complexity of post-transcriptional networks in vivo. The co-downregulation suggests that miR-335-5p and *SFTPD* may be subject to a common upstream suppressive regulator in CRC, or that the *SFTPD*/miR-335-5p/lnc-HNRNPUL2 axis operates within a broader network involving additional undiscovered components [70].

ROC curve analysis of the biomarker panel in CRC patients compared with the non-malignant group revealed that lnc-HNRNPUL2 possessed a significantly higher sensitivity for the CRC patients than the traditional biomarkers CEA and CA19.9. In addition, logistic regression analysis revealed that HNRNPUL2 and *SFTPD* mRNA were strong predictive factors for the diagnosis of CRC.

In the CRC, we hypothesized that the *SFTPD*/miR-335-5p/lnc-HNRNPUL2 xis is crucial in CRC pathogenesis. Firstly, the upregulation of the lnc-HNRNPUL2 acting as propsed regulatory axis for miR-335-5p in CRC based on the in silico analysis and previous evidence from other studies [60]. Secondly, downregulated miR-335-5p inhibits its direct interaction with the *SFTPD* promoter, preventing the recruitment of transcription factors and RNA-Polymerase-II on the *SFTPD* promoter, decreasing its expression. Thirdly, the down-regulation of the *SFTPD* may lead to enhancing T-cell proliferation and inflammatory cytokines, inducing the release of TGF-B & TNF-α that stimulate tumor cell proliferation, invasion, and metastasis, as well as increasing white blood cells, TGF-β as an integrative node in the proposed network. The significant elevation of TGF-β in CRC serum is mechanistically coherent within the proposed axis. First, miR-335-5p is a known direct repressor of TGF-β1 (as validated in miRTarBase and supported by literature in other cancers). Thus, the observed downregulation of miR-335-5p in CRC would be expected to derepress TGF-β signaling. Second, TGF-β is a potent suppressor of anti-tumor immunity and a driver of epithelial-mesenchymal transition (EMT). Concurrently, *SFTPD* has been shown to antagonize TGF-β-induced EMT and promote immune surveillance in pancreatic and lung cancer models. Therefore, we propose a model wherein the downregulation of the *SFTPD*/miR-335-5p axis removes a dual brake on TGF-β activity: (i) via reduced miRNA-mediated repression and (ii) via loss of *SFTPD*-mediated antagonism of TGF-β signaling. This creates a permissive environment for TGF-β-driven immune evasion and tumor progression [65, 66]; Inducing p38 MAPK activation via the interaction of the collagen part of the SP-D structure with immune cells and hence increasing CRC cells’ tumorigenicity [67] (Fig. 1B). Fourth, the Activation of TGF-β stimulates tumor growth, invasion, and metastasis [68].

It is important to distinguish between the direct regulatory interactions within the core RNA axis (e.g., miRNA-mRNA binding) and the functional and correlative link to TGF-β. While TGF-β is a downstream target of miR-335-5p and is functionally opposed by *SFTPD*, its serum level serves here as a supportive, clinically measurable indicator of the immunosuppressive pathway activated upon dysregulation of the *SFTPD*/miR-335-5p/lnc-HNRNPUL2 axis.

**Fig. 7:**
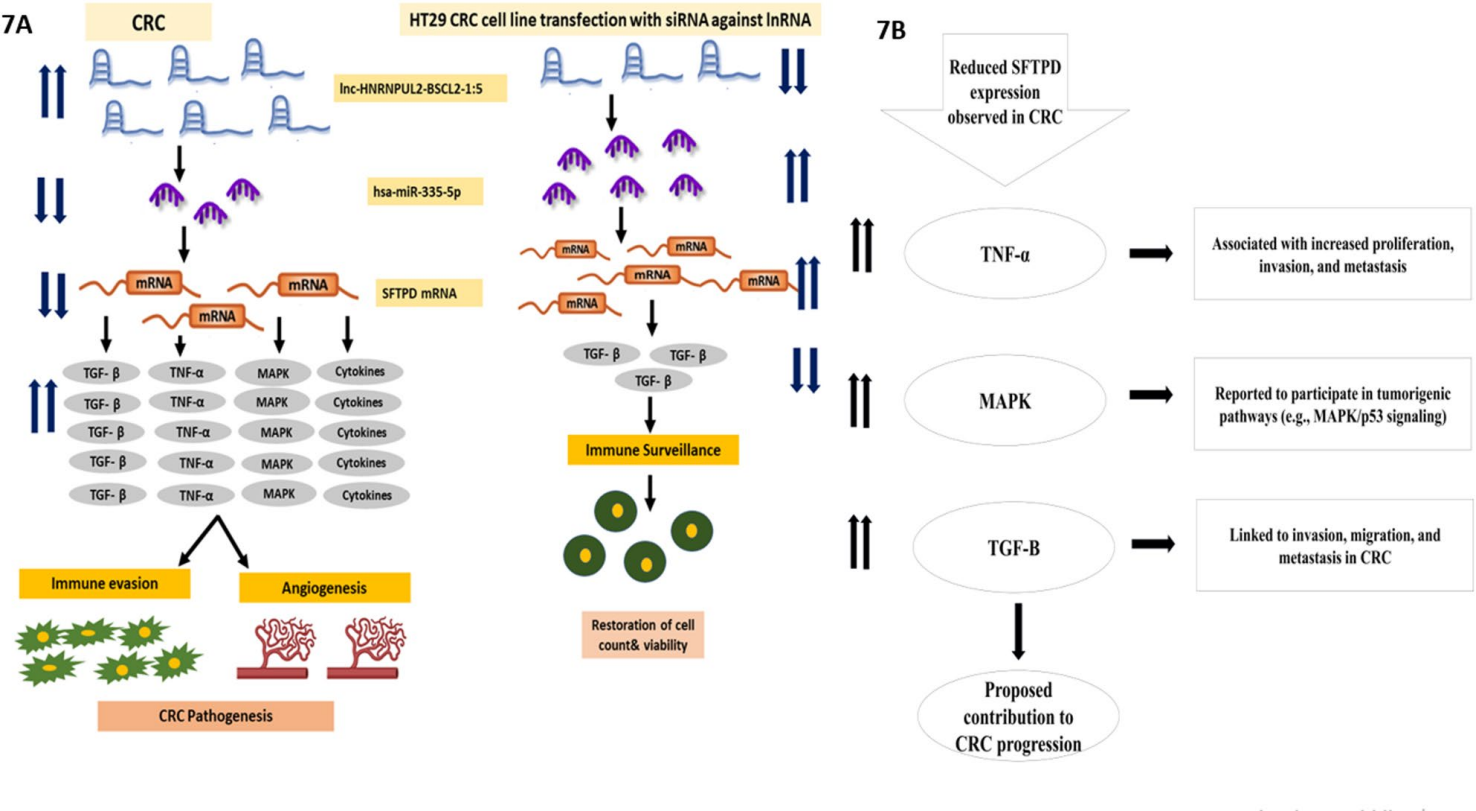
Proposed regulatory model based on bioinformatic prediction and correlative findings. **A** Proof of concept of the study hypothesis. Reduced SFTPD expression observed in CRC is shown in association with increased TNF-α, MAPK, and TGF-β signaling, which have been linked to proliferation, invasion, and metastasis, potentially contributing to CRC progression. **B** The Proposed mechanisms for *SFTPD-*mediated immune evasion in the CRC. In CRC and HT29 cells, increased lnc-HNRNPUL2 expression together with reduced miR-335-5p and *SFTPD* levels is observed in parallel with features consistent with immune evasion and angiogenesis. Silencing of lnc-HNRNPUL2 in HT29 cells was accompanied by reciprocal changes in miR-335-5p and *SFTPD* expression, supporting a possible regulatory relationship within this proposed axis

Collectively, based on our integrated bioinformatics, clinical, and functional data, the present study support a tumor-suppressive role for *SFTPD* in CRC: its downregulation in CRC sera, its negative correlation with oncogenic markers (HNRNPUL2 and TGF-β1), and its restoration upon lnc-HNRNPUL2 knockdown in HT29 cells are all consistent with *SFTPD* acting as a suppressor of CRC progression through immune surveillance mechanisms (Fig. 7A& B). This model aligns with established roles of *SFTPD* in immune regulation and TGF-β in CRC pathogenesis [24, 40].

This study has several limitations. Foremost, larger, well-designed cohorts are required to substantiate our findings and improve their generalizability. Further in-depth mechanistic studies, including luciferase reporter assays, RNA immunoprecipitation, or pull-down experiments, are needed to confirm the direct binding interactions within the *SFTPD*/miR-335-5p/lnc-HNRNPUL2 axis. Additionally, in vitro functional validation was restricted to a single CRC cell line (HT29), which may not capture the molecular heterogeneity of CRC. Future studies should extend functional analyses to additional CRC models (e.g., HCT116, SW480, Caco-2) representing distinct molecular subtypes and functional experiments such as Transwell migration/invasion, apoptosis, and EMT marker analysis to fully characterize the mechanistic role of the *SFTPD*/miR-335-5p/lnc-HNRNPUL2 axis in CRC progression. Also, the precise regulatory relationship between miR-335-5p and *SFTPD* mRNA remains to be experimentally defined. While our bioinformatics analysis predicts targeting, and our clinical data show a significant correlation, direct evidence of repression or any non-canonical regulation requires validation through luciferase reporter assays and functional rescue experiments in future studies. The short follow-up period (14 ± 6.9 months) precludes meaningful overall survival analysis. While we identified significant associations between low miR-335-5p and metastasis using Cox regression, these findings are preliminary. True prognostic validation requires studies with standard CRC follow-up durations (3–5 years) and larger cohorts to adequately power survival analyses. The observed BMI differences between groups, though adjusted for statistically, could reflect underlying metabolic differences that influence biomarker expression. Future studies should employ stricter matching criteria or larger samples to facilitate more comprehensive adjustment. While the combined panel achieved maximal sensitivity, its moderate specificity highlights the inherent trade-off between detection and false positives. Importantly, the multivariate model demonstrated improved diagnostic balance, suggesting greater suitability for clinical application. Future studies must prioritize validation in a large, multicenter, prospective, independent cohort.

## Conclusion

The *SFTPD* panel, including *SFTPD*/miR-335-5p/lnc-HNRNPUL2, and TGF-β, plays an important role in the development of CRC by mediating tumor evasion from immune surveillance via proposed lnc-HNRNPUL2 regulatory RNA axis. While the *SFTPD* panel demonstrates strong discriminating utility, its prognostic significance requires validation in studies with longer follow-up. Our preliminary findings suggest that low miR-335-5p may be associated with metastasis, warranting further investigation.

## Supplementary Information

Supplementary Material 1.

Supplementary Material 2.

## Data Availability

Data are available from the corresponding author on reasonable request.

## Abbreviations

CA19.9: Carbohydrate antigen 19.9
CA125: Cancer antigen 125
CA72-4: Cancer antigen 72 − 4
CDK6: Cyclin-dependent kinase 6
CEA: Carcinoembryonic antigen
CRC: Colorectal cancer
ceRNA: Competing endogenous RNA
Ct: Threshold cycle
DEGs: Differentially expressed genes
ELISA: Enzyme-linked immunosorbent assay
EMT: Epithelial-to-mesenchymal transition
GEO: Gene Expression Omnibus
GO: Gene ontology
FDR: False discovery rate
KEGG: Kyoto Encyclopedia of Genes and Genomes
Lnc-RNAs: Long noncoding RNAs
MRNAs: Messenger RNAs
MiRNAs: microRNAs
NSCLC: Non-small cell lung cancer
ROC: Receiver operating characteristic
SFTPD: Surfactant protein D
TAG 72: Tumor-associated glycoprotein 72
TGF-β: Transforming growth factor-β
TNF-α: Tumor necrosis factor-α
TPM: Transcripts per million
VEGF: Vascular Endothelial Growth Factor
ROCK1: Rho-associated coiled-coil containing protein
siRNA: Small interfering RNA
SP1: Specificity protein 1
T2DM: Type 2 diabetes mellitus
TLR4: Toll-Like Receptor 4
LPS: Lipopolysaccharides
UTR: Untranslated region

## References

[1] Talaat IM, Elemam NM, Saber-Ayad M. Complement System: An Immunotherapy Target in Colorectal Cancer. Front Immunol. 2022;13:1–12. 10.3389/fimmu.2022.810993. 10.3389/fimmu.2022.810993 PMC8841337 35173724

[2] Klimeck L, Heisser T, Hoffmeister M, Brenner H. Colorectal cancer: A health and economic problem. Best Pract Res Clin Gastroenterol. Oct. 2023;66:101839. 10.1016/J.BPG.2023.101839. 10.1016/j.bpg.2023.101839 37852707

[3] Dekker E, Tanis PJ, Vleugels JLA, Kasi PM, Wallace MB. Colorectal cancer. Lancet (London England). Oct. 2019;394:1467–80. 10.1016/S0140-6736(19)32319-0. 10.1016/S0140-6736(19)32319-0 31631858

[4] Bader El NG, Din et al. Feb., MicroRNAs expression profiling in Egyptian colorectal cancer patients., IUBMB Life, vol. 72, no. 2, pp. 275–284, 2020, 10.1002/iub.2164. 10.1002/iub.2164 31512372

[5] Liu N, Jiang F, Chen Z. A Preliminary Study on the Pathogenesis of Colorectal Cancer by Constructing a Hsa-circRNA-0067835-miRNA-mRNA Regulatory Network. Onco Targets Ther. 2021;14:4645–58. 10.2147/OTT.S319300. 34511934 10.2147/OTT.S319300 PMC8418363

[6] Schmitt M, Greten FR. The inflammatory pathogenesis of colorectal cancer, Nat. Rev. Immunol. 2021 2110, vol. 21, no. 10, pp. 653–667, Apr. 2021. 10.1038/s41577-021-00534-x. 10.1038/s41577-021-00534-x 33911231

[7] Grasso CS, et al. Genetic mechanisms of immune evasion in colorectal cancer. Cancer Discov. 2018;8(6):730–49. 10.1158/2159-8290.CD-17-1327. 29510987 10.1158/2159-8290.CD-17-1327 PMC5984687

[8] Agüera-Sánchez A, Peña-Ros E, Martínez-Martínez I, García-Molina F. Comprehensive Landscape of Diagnostic, Prognostic and Predictive Biomarkers in Colorectal Cancer: From Genomics to Multi-Omics Integration in Precision Medicine, J. Pers. Med. 2026, Vol. 16, vol. 16, no. 1, p. 48, Jan. 2026. 10.3390/jpm16010048. 10.3390/jpm16010048 PMC12842678 41590540

[9] Guerrouahen BS, Maccalli C, Cugno C, Rutella S, Akporiaye ET. Reverting Immune Suppression to Enhance Cancer Immunotherapy. Front Oncol. January, 2020;9. 10.3389/fonc.2019.01554. 10.3389/fonc.2019.01554 PMC6985581 32039024

[10] Mangogna A et al. Pathological significance and prognostic value of surfactant protein D in cancer, Front. Immunol., vol. 9, no. AUG, pp. 1–12, 2018, 10.3389/fimmu.2018.01748. 10.3389/fimmu.2018.01748 PMC6088209 30127783

[11] Kumar J, et al. Surfactant Protein D as a Potential Biomarker and Therapeutic Target in Ovarian Cancer. Front Oncol. 2019;9:1–14. 10.3389/fonc.2019.00542. 31338320 10.3389/fonc.2019.00542 PMC6629871

[12] Kaur A, Riaz MS, Murugaiah V, Varghese PM, Singh SK, Kishore U. A Recombinant fragment of human surfactant protein D induces apoptosis in pancreatic cancer cell lines via fas-mediated pathway, Front. Immunol., vol. 9, no. JUN, 2018, 10.3389/fimmu.2018.01126. 10.3389/fimmu.2018.01126 PMC5994421 29915574

[13] Vieira LM, Jorge NAN, de Sousa JB, Setubal JC, Stadler PF, Walter MEMT. Competing Endogenous RNA in Colorectal Cancer: An Analysis for Colon, Rectum, and Rectosigmoid Junction, Front. Oncol., vol. 11, no. June, pp. 1–11, 2021, 10.3389/fonc.2021.681579. 10.3389/fonc.2021.681579 PMC8222815 34178670

[14] Pi YN, Qi WC, Xia BR, Lou G, Jin WL. Long Non-Coding RNAs in the Tumor Immune Microenvironment: Biological Properties and Therapeutic Potential, Front. Immunol., vol. 12, no. July, pp. 1–13, 2021, 10.3389/fimmu.2021.697083. 10.3389/fimmu.2021.697083 PMC8290853 34295338

[15] Wu M, Fu P, Qu L, Liu J, Lin A. Long Noncoding RNAs, New Critical Regulators in Cancer Immunity, Front. Oncol., vol. 10, no. October, pp. 1–7, 2020, 10.3389/fonc.2020.550987. 10.3389/fonc.2020.550987 PMC7662117 33194608

[16] Wang Y, Fu J, Yang L, Liang Z. Long non–coding RNA SNHG20 promotes colorectal cancer cell proliferation, migration and invasion via miR–495/STAT3 axis. Mol Med Rep. Jan. 2021;23(1). 10.3892/mmr.2020.11669. 10.3892/mmr.2020.11669 PMC7705999 33179110

[17] Li C et al. Jan., Long intergenic non-coding RNA LINC00485 exerts tumor-suppressive activity by regulating miR-581/EDEM1 axis in colorectal cancer., Aging (Albany. NY)., vol. 13, no. 3, pp. 3866–3885, 2021, 10.18632/aging.202354. 10.18632/aging.202354 PMC7906134 33461166

[18] Xiao Z, et al. Function and mechanisms of microRNA–20a in colorectal cancer (Review). Exp Ther Med. 2020;1605–16. 10.3892/etm.2020.8432. 10.3892/etm.2020.8432 PMC7027132 32104211

[19] Pidíkova P, Reis R, Herichova I. miRNA Clusters with Down-Regulated Expression in Human Colorectal Cancer and Their Regulation., Int. J. Mol. Sci., vol. 21, no. 13, Jun. 2020. 10.3390/ijms21134633. 10.3390/ijms21134633 PMC7369991 32610706

[20] Correll KA, Edeen KE, Zemans RL, Redente EF, Mikels-Vigdal A, Mason RJ. TGF beta inhibits expression of SP-A, SP-B, SP-C, but not SP-D in human alveolar type II cells. Biochem Biophys Res Commun. 2018;499(4). 10.1016/j.bbrc.2018.04.003. 10.1016/j.bbrc.2018.04.003 PMC6204198 29621540

[21] Kaur A, Riaz MS, Singh SK, Kishore U. Human surfactant protein D suppresses epithelial-to-mesenchymal transition in pancreatic cancer cells by downregulating TGF-β, Front. Immunol., vol. 9, no. AUG, pp. 1–13, 2018. 10.3389/fimmu.2018.01844. 10.3389/fimmu.2018.01844 PMC6104167 30158928

[22] Ding Y, Li W, Wang K, Xu C, Hao M, Ding L. Perspectives of the Application of Liquid Biopsy in Colorectal Cancer. 2020. 10.1155/2020/6843180. 10.1155/2020/6843180 PMC7085834 32258135

[23] Heidrich I, Abdalla TSA, Reeh M, Pantel K. Clinical applications of circulating tumor cells and circulating tumor DNA as a liquid biopsy marker in colorectal cancer. 2021. 10.3390/cancers13184500. 10.3390/cancers13184500 PMC8469158 34572727

[24] Hu M et al. Circulating tumor cells in colorectal cancer in the era of precision medicine. 2022. 10.1007/s00109-021-02162-3. 10.1007/s00109-021-02162-3 PMC8770420 34802071

[25] Vafaei S, Fattahi F, Ebrahimi M, Janani L, Shariftabrizi A, Madjd Z. Common molecular markers between circulating tumor cells and blood exosomes in colorectal cancer: A systematic and analytical review. Cancer Manag Res. 2019;11. 10.2147/CMAR.S219699. 10.2147/CMAR.S219699 PMC6768129 31576171

[26] Egner JR, Cancer Staging AJCC, Manual. JAMA, vol. 304, no. 15, p. 1726, 2010, 10.1001/jama.2010.1525.

[27] Alhopuro P, et al. Candidate driver genes in microsatellite-unstable colorectal cancer. Int J cancer. Apr. 2012;130(7):1558–66. 10.1002/IJC.26167. 10.1002/ijc.26167 21544814

[28] Hong Y, Kok SH, Kong WE, Peh YC. A susceptibility gene set for early onset colorectal cancer that integrates diverse signaling pathways: implication for tumorigenesis. Clin Cancer Res. Feb. 2007;13(4):1107–14. 10.1158/1078-0432.CCR-06-1633. 10.1158/1078-0432.CCR-06-1633 17317818

[29] Tsukamoto S et al. Apr., Clinical significance of osteoprotegerin expression in human colorectal cancer, Clin. Cancer Res., vol. 17, no. 8, pp. 2444–2450, 2011, 10.1158/1078-0432.CCR-10-2884. 10.1158/1078-0432.CCR-10-2884 21270110

[30] Du W, et al. MiR-335-5p inhibits TGF-β1-induced epithelial-mesenchymal transition in non-small cell lung cancer via ROCK1. Respir Res. 2019;20(1):1–11. 10.1186/s12931-019-1184-x. 31638991 10.1186/s12931-019-1184-x PMC6805547

[31] Lynch J, et al. MiRNA-335 suppresses neuroblastoma cell invasiveness by direct targeting of multiple genes from the non-canonical TGF-β signalling pathway. Carcinogenesis. 2012;33(5). 10.1093/carcin/bgs114. 10.1093/carcin/bgs114 PMC3334516 22382496

[32] Jung G, Hernández-Illán E, Moreira L, Balaguer F, Goel A. Epigenetics of colorectal cancer: biomarker and therapeutic potential., Nat. Rev. Gastroenterol. Hepatol., vol. 17, no. 2, pp. 111–130, Feb. 2020, 10.1038/s41575-019-0230-y. 10.1038/s41575-019-0230-y PMC7228650 31900466

[33] Quintero E, Castells A, Bujanda L. Colonoscopy versus Fecal Immunochemical Testing in Colorectal-Cancer Screening. Gastroenterol Endosc. 2012;54(4):1510. 10.11280/gee.54.1510. 10.1056/NEJMoa1108895 22356323

[34] Roy S, et al. Diagnostic efficacy of circular RNAs as noninvasive, liquid biopsy biomarkers for early detection of gastric cancer. Mol Cancer. 2022;21(1). 10.1186/s12943-022-01527-7. 10.1186/s12943-022-01527-7 PMC8826675 35139874

[35] He JH et al. The Role of Liquid Biopsy Analytes in Diagnosis, Treatment and Prognosis of Colorectal Cancer. 2022. 10.3389/fendo.2022.875442. 10.3389/fendo.2022.875442 PMC9279561 35846270

[36] Raza A et al. Dynamic liquid biopsy components as predictive and prognostic biomarkers in colorectal cancer. 2022. 10.1186/s13046-022-02318-0. 10.1186/s13046-022-02318-0 PMC8922757 35292091

[37] Baassiri A, Nassar F, Mukherji D, Shamseddine A, Nasr R, Temraz S. Exosomal non coding RNA in LIQUID biopsies as a promising biomarker for colorectal cancer, 2020. 10.3390/ijms21041398. 10.3390/ijms21041398 PMC7073025 32092975

[38] Tieng FYF, Abu N, Lee LH, Ab Mutalib NS. Microsatellite instability in colorectal cancer liquid biopsy—current updates on its potential in non-invasive detection, prognosis and as a predictive marker. 2021. 10.3390/diagnostics11030544. 10.3390/diagnostics11030544 PMC8003257 33803882

[39] Vinod R, Mahran R, Routila E, Leivo J, Pettersson K, Gidwani K. Nanoparticle-aided detection of colorectal cancer-associated glycoconjugates of extracellular vesicles in human serum. Int J Mol Sci. 2021;22(19). 10.3390/ijms221910329. 10.3390/ijms221910329 PMC8508761 34638669

[40] Jian Y, et al. Current Advance of Immune Evasion Mechanisms and Emerging Immunotherapies in Renal Cell Carcinoma. Front Immunol. 2021;12:1–21. 10.3389/fimmu.2021.639636. 10.3389/fimmu.2021.639636 PMC7985340 33767709

[41] Guo L, et al. Construction and Analysis of a ceRNA Network Reveals Potential Prognostic Markers in Colorectal Cancer. Front Genet. May, 2020;11. 10.3389/fgene.2020.00418. 10.3389/fgene.2020.00418 PMC7228005 32457800

[42] Hoffmann-Petersen B, et al. Association of serum surfactant protein D and SFTPD gene variants with asthma in Danish children, adolescents, and young adults. Immun Inflamm Dis. 2021;no May 2021:189–200. 10.1002/iid3.560. 10.1002/iid3.560 PMC8767520 34780682

[43] Murugaiah V et al. Hyaluronic Acid Present in the Tumor Microenvironment Can Negate the Pro-apototic Effect of a Recombinant Fragment of Human Surfactant Protein D on Breast Cancer Cells, Front. Immunol., vol. 11, no. July, pp. 1–17, 2020, 10.3389/fimmu.2020.01171. 10.3389/fimmu.2020.01171 PMC7360846 32733438

[44] Pandit H, et al. Surfactant protein D induces immune quiescence and apoptosis of mitogen-activated peripheral blood mononuclear cells. Immunobiology. 2016;221(2):310–22. 10.1016/j.imbio.2015.10.004. 26563748 10.1016/j.imbio.2015.10.004

[45] Sorensen GL, et al. Association between the surfactant protein D (SFTPD) gene and subclinical carotid artery atherosclerosis. Atherosclerosis. Mar. 2016;246:7–12. 10.1016/J.ATHEROSCLEROSIS.2015.12.037. 10.1016/j.atherosclerosis.2015.12.037 26748346

[46] Madan T, Reid KBM, Singh M, Sarma PU, Kishore U. Susceptibility of Mice Genetically Deficient in the Surfactant Protein (SP)-A or SP-D Gene to Pulmonary Hypersensitivity Induced by Antigens and Allergens of Aspergillus fumigatus. J Immunol. 2005;174(11). 10.4049/jimmunol.174.11.6943. 10.4049/jimmunol.174.11.6943 15905537

[47] Tajima Y, et al. Association of surfactant protein D with pulmonary metastases from colon cancer. Oncol Lett. 2020;20(6). 10.3892/OL.2020.12185. 10.3892/ol.2020.12185 PMC7583848 33123238

[48] Zhang L, Meng Q, Yepuri N, Wang G, Xi X, Cooney RN. Surfactant Proteins-A and-D Attenuate LPS-Induced Apoptosis in Primary Intestinal Epithelial Cells (IECs). Shock. 2018;49(1). 10.1097/SHK.0000000000000919. 10.1097/SHK.0000000000000919 28591009

[49] Mahajan L, et al. Human surfactant protein D alters oxidative stress and HMGA1 expression to induce p53 apoptotic pathway in eosinophil leukemic cell line. PLoS ONE. 2013;8(12). 10.1371/journal.pone.0085046. 10.1371/journal.pone.0085046 PMC3877357 24391984

[50] Leth-Larsen R, et al. A Common Polymorphism in the SFTPD Gene Influences Assembly, Function, and Concentration of Surfactant Protein D. J Immunol. 2005;174(3). 10.4049/jimmunol.174.3.1532. 10.4049/jimmunol.174.3.1532 15661913

[51] Glapa-Nowak A, et al. Genetic variants of dmbt1 and sftpd and disease severity in paediatric inflammatory bowel disease—a polish population-based study. Children. 2021;8(11). 10.3390/children8110946. 10.3390/children8110946 PMC8618964 34828659

[52] Tanaka M, et al. Genetic variants in surfactant, pulmonary-associated protein D (SFTPD) and Japanese susceptibility to ulcerative colitis. Inflamm Bowel Dis. 2009;15(6). 10.1002/ibd.20936. 10.1002/ibd.20936 19340882

[53] Yuan W, et al. Comprehensive analysis of lncRNA-associated ceRNA network in colorectal cancer. Biochem Biophys Res Commun. 2019;508(2):374–9. 10.1016/j.bbrc.2018.11.151. 30503344 10.1016/j.bbrc.2018.11.151

[54] Zhang Y, et al. Long non-coding RNA H19 promotes colorectal cancer metastasis via binding to hnRNPA2B1. J Exp Clin Cancer Res. 2020;39(1):1–15. 10.1186/s13046-020-01619-6. 32698890 10.1186/s13046-020-01619-6 PMC7412843

[55] Jiang H, et al. Long noncoding rna crnde stabilized by hnrnpul2 accelerates cell proliferation and migration in colorectal carcinoma via activating ras/mapk signaling pathways. Cell Death Dis. 2017;8(6). 10.1038/cddis.2017.258. 10.1038/cddis.2017.258 PMC5520914 28594403

[56] Ye L et al. Functions and targets of mir-335 in cancer, Onco. Targets. Ther., vol. 14, no. May, pp. 3335–3349, 2021, 10.2147/OTT.S305098. 10.2147/OTT.S305098 PMC8144171 34045870

[57] KrishnaPriya S, Nair PS, Bhalla P, Karunagaran D, Suraishkumar GK. Shear stress and microRNAs for better metastatic cancer management. Biotechnol Prog. Jan. 2024;40(1). 10.1002/btpr.3396. 10.1002/btpr.3396 37843824

[58] Duan L, et al. Molecular mechanisms and clinical implications of miRNAs in drug resistance of colorectal cancer. Ther Adv Med Oncol. 2020;12:1758835920947342. 10.1177/1758835920947342. 32922521 10.1177/1758835920947342 PMC7450467

[59] Jia Q, et al. Circular RNA 0007255 regulates the progression of breast cancer through miR-335-5p/SIX2 axis. Thorac Cancer. 2020;11(3):619–30. 10.1111/1759-7714.13306. 31962380 10.1111/1759-7714.13306 PMC7049509

[60] Liang H, Zhang C, Guan H, Liu J, Cui Y. LncRNA DANCR promotes cervical cancer progression by upregulating ROCK1 via sponging miR-335-5p. J Cell Physiol. 2019;234(5):7266–78. 10.1002/jcp.27484. 30362591 10.1002/jcp.27484

[61] Gao Y, et al. miR-335-5p suppresses gastric cancer progression by targeting MAPK10. Cancer Cell Int. 2021;21(1):1–12. 10.1186/s12935-020-01684-z. 33482821 10.1186/s12935-020-01684-z PMC7821696

[62] Zhang D, Yang N. MiR-335-5p inhibits cell proliferation, migration and invasion in colorectal cancer through downregulating LDHB. J B U ON. 2019;24(3):1128–36. 31424671

[63] Luo L et al. miR-335-5p targeting ICAM-1 inhibits invasion and metastasis of thyroid cancer cells, Biomed. Pharmacother., vol. 106, no. July, pp. 983–990, 2018, 10.1016/j.biopha.2018.07.046. 10.1016/j.biopha.2018.07.046 30119270

[64] Oner M, et al. Future aspects of CDK5 in prostate cancer: From pathogenesis to therapeutic implications. Int J Mol Sci. 2019;20(16). 10.3390/ijms20163881. 10.3390/ijms20163881 PMC6720211 31395805

[65] Lu Y, Li Y, Li G, Lu H. Identification of potential markers for type 2 diabetes mellitus via bioinformatics analysis. Mol Med Rep. 2020;22(3). 10.3892/mmr.2020.11281. 10.3892/mmr.2020.11281 PMC7411335 32705173

[66] Wang H, et al. Effect of miR-335 upregulation on the apoptosis and invasion of lung cancer cell A549 and H1299. Tumor Biol. 2013;34(5). 10.1007/s13277-013-0878-9. 10.1007/s13277-013-0878-9 23740614

[67] O’Brien J, Hayder H, Zayed Y, Peng C. Overview of microRNA biogenesis, mechanisms of actions, and circulation, Front. Endocrinol. (Lausanne)., vol. 9, no. AUG, pp. 1–12, 2018, 10.3389/fendo.2018.00402. 10.3389/fendo.2018.00402 PMC6085463 30123182

[68] Lee S, et al. A post-transcriptional program of chemoresistance by AU-rich elements and TTP in quiescent leukemic cells. Genome Biol. 2020;21(1). 10.1186/s13059-020-1936-4. 10.1186/s13059-020-1936-4 PMC7011231 32039742

[69] Kay M, Soltani BM, Aghdaei FH, Ansari H, Baharvand H. Hsa-miR-335 regulates cardiac mesoderm and progenitor cell differentiation. Stem Cell Res Ther. 2019;10(1). 10.1186/s13287-019-1249-2. 10.1186/s13287-019-1249-2 PMC6595595 31248450

[70] Quéméner AM, et al. Non-canonical miRNA-RNA base-pairing impedes tumor suppressor activity of miR-16. Life Sci Alliance. Dec. 2022;5(12). 10.26508/lsa.202201643. 10.26508/lsa.202201643 PMC9553902 36202613

