# Supplementary material for: Role of SFTPD/miR-335-5p/lnc-HNRNPUL2 axis in colorectal cancer: in silico characterization and clinical validation
Source: BMC Cancer. 2026 Mar 26;26:459. doi: 10.1186/s12885-026-15833-6 (PMC13063474; doi:10.1186/s12885-026-15833-6)
Supplement: Supplementary file 2 — Supplementary Material 2. [file 12885_2026_15833_MOESM2_ESM.docx]

**Supplementary Tables:**

**Table S2. Details of the GSE21510 datasets retrieved from the GEO database.**

| Accession number | Platform | Experiment Title | Organism | Experimental Design |
| --- | --- | --- | --- | --- |
| GSE21510 | GPL570 [HG-U133_Plus_2] Affymetrix Human Genome U133 Plus 2.0 Array. | Clinical Significance of Osteoprotegerin Expression in Human Colorectal Cancer | Homo sapiens | A total of 104 patients were assigned to the microarray study, including 13 patients with stage I, 37 patients with stage II, 34 patients with stage III, and 20 patients with stage IV disease.  19 patients with noncancerous tissues from 25 patients using homogenized tissues. This study was held in Tokyo Medical and Dental University Hospital in Jaban. |
| GSE4107 | [GPL570](https://www.ncbi.nlm.nih.gov/geo/query/acc.cgi?acc=GPL570)[HG-U133_Plus_2] Affymetrix Human Genome U133 Plus 2.0 Array | \| Expression profiling  in early onset  colorectal cancer \| \| --- \| \|  \| | Homo sapiens | RNA extracted from colonic mucosa of healthy controls(10samples) and patients(12samples) were analyzed using GeneChip U133-Plus 2.0 Array. Patients and controls were age- (50 or less), ethnicity- (Chinese) and tissue-matched. The study was held at the Singapore General Hospital. |
| GSE24514 | \| \|  \| \| --- \|  \| [GPL96](https://www.ncbi.nlm.nih.gov/geo/query/acc.cgi?acc=GPL96) \| [HG-U133A] Affymetrix Human Genome U133A Array \| \| --- \| --- \| \| \| --- \| --- \| --- \| --- \| | \| Expression data  from human MSI  colorectal cancer  and normal colonic  mucosa \| \| --- \| \|  \| | Homo sapiens | Expression profiling of 34 MSI colorectal cancers and 15 normal colonic mucosa. Comparison of malignant and healthy tissue. The cancerous tissues were obtained from the American Type Culture Collection (ATCC) and the European Collection of Cell Cultures(ECACC) and the normal tissues and the study was held on Finland. |

**Table S3: Bioinformatics Rationale and Selection Criteria for the SFTPD/miR-335-5p/lncHNRNPUL2 Axis in CRC**

|  | **Type** | **Selection Rationale** | **Bioinformatics Database(s)** | **Cutoff / Criteria** | **validation** | **visualization** | **Significant Results** | **Supporting Literature** |
| --- | --- | --- | --- | --- | --- | --- | --- | --- |
| **SFTPD** | mRNA | Immune surveillance role; downregulated in CRC; functional relevance to TGF-β signaling and immune evasion. | **GEO (GSE21510, GSE24514, GSE4107)** | Normalization: RMA/Quantile  • DEG: limma, FDR<0.05, \|log₂FC\|≥1  • Intersection: ≥2/3 datasets | Expression ATLAS (TPM validation) UniProt (GO:0006955 immune response) | Volcano plots (Fig 1a-c) Heatmap (Fig S1e) | Downregulated in CRC across all datasets | Kaur et al., 2018 (Front Immunol); Mangogna et al., 2018 (Front Immunol) |
|  |  | Tissue-specific expression validation in CRC. | **Expression ATLAS** | TPM-based expression heatmap |  |  | Lower expression in CRC vs. normal (Fig S1) | – |
|  |  | Functional annotation and pathway relevance. | **UniProt** | GO terms: immune response, surfactant homeostasis |  |  | GO:0006955 (immune response), GO:0043129 (surfactant homeostasis) (Fig S2) | – |
| **miR-335-5p** | miRNA | Predicted to target SFTPD 3′UTR; involved in TGF-β signaling and cancer progression. | **miRBase, DIANA-TarBase, miRTarBase** | • Binding: TargetScan score >0.7 • Validation: miRTarBase experimental support • Pathway: DIANA-miRPath, FDR<0.05 | GeneCards (colon expression) Literature: Tavazoie et al., 2008 | Binding site diagram (Fig S3) KEGG pathway map (Fig S4) | Validated interaction with SFTPD (Fig S3) | - Lu et al., 2020 (Mol Med Rep); Du et al., 2019 (Respir Res) - **Tavazoie SF, Alarcón C, Oskarsson T, Padua D, Wang Q, Bos PD, Gerald WL, Massagué J. Endogenous human microRNAs that suppress breast cancer metastasis. Nature. 2008 Jan 10;451(7175):147-52. doi: 10.1038/nature06487** |
|  |  | KEGG pathway enrichment in CRC-related pathways. | **DIANA-miRPath v3.0** | Padj<0.05*Padj*​<0.05, Fisher’s exact test |  |  | Enriched in TGF-β signaling, CRC pathways (Fig S4) | – |
|  |  | Tissue expression in colon. | **GeneCards** | Expression score > 1 (moderate–high) |  |  | Expressed in colon tissue (Fig S5) | – |
| **HNRNPUL2** | lncRNA | Predicted to sponge miR-335-5p; upregulated in CRC; linked to RNA stability and cancer. | **InCeDB, LncBook 2.0** | • Interaction: InCeDB score >0.8, P<0.05 • Expression: LncBook TPM, \|log₂FC\|≥1 • Network: Ensembl direct interactions | CRC expression validation (Fig S7) Literature: Jiang et al., 2017 | Interaction network (Fig S6) Expression boxplot (Fig S7) | Binds miR-335-5p (Fig S6); upregulated in CRC (Fig S7) | Jiang et al., 2017 (Cell Death Dis) |
|  |  | Interaction with TGF-β1/β2. | **Ensembl** | Direct interaction evidence |  |  | Physical interaction with TGF-β1/β2 (Fig S9) | – |
| **TGF-β** | Cytokine | Validated target of miR-335-5p; key player in immune evasion and EMT in CRC. | **miRTarBase** | Experimentally validated |  |  | Direct targeting by miR-335-5p (Fig S8) | Kaur et al., 2018 (Front Immunol); Du et al., 2019 (Respir Res) |
| **Combined Panel** | RNA panel | Enriched in immune-related GO terms and cancer pathways. | **Enrichr** | Padj<0.05*Padj*​<0.05 |  |  | Enriched in immune response, extracellular exosomes, TGF-β signaling (Fig S10) | – |

**Excluded Data / Justification for Omission**

- **Other miRNAs targeting SFTPD:** Multiple miRNAs were predicted (e.g., miR-155-5p, miR-146a-5p) but excluded due to lower binding scores or lack of CRC-specific literature support.
- **Other lncRNAs interacting with miR-335-5p:** Candidates such as MALAT1 and H19 were excluded because they are widely studied in CRC, and we prioritized novel, less-characterized lncRNAs.
- **Non-immune-related DEGs from GEO:** Genes unrelated to immune surveillance or TGF-β signaling were filtered out to maintain focus on the proposed axis.
- **Low-confidence bioinformatics predictions:** Interactions with prediction scores below 0.7 or lacking cross-database validation were excluded to ensure robustness.

**Table S4.: List of primers used for qPCR.**

| Primer name | GeneGlobe ID |
| --- | --- |
| ***SFTPD mRNA*** | NM_003019 |
| GAPDH | NM_002046 |
| hsa-miR-335-5p | MIMAT0000765 |
| lnc-HNRNPUL2 | ENST00000540127.1(HNRNPUL2-202) |
| SNORD-68 | SBH0614997 |

**Table S5.: Spearman's rho correlation between the expressions of lncRNA HNRNPUL2, SFTPD mRNA, and hsa-miR-335-5p between (A) All groups. (B) The control and the CRC groups. (C) The control and the Benign groups. (D) The CRC group only.**

| (A)   \| **Correlations** \| \| \| \| \| \| \| --- \| --- \| --- \| --- \| --- \| --- \| \|  \| \| \| lnc-HNRNPUL2 \| SFTPD mRNA \| hsa-miR-335-5p \| \| Spearman's rho \| lnc-HNRNPUL2 \| Correlation Coefficient \| 1.000 \| -.517^**^ \| -.532^**^ \| \| Sig. (2-tailed) \| . \| .000 \| .000 \| \| N \| 152 \| 152 \| 152 \| \| SFTPD mRNA \| Correlation Coefficient \| -.517^**^ \| 1.000 \| .531^**^ \| \| Sig. (2-tailed) \| .000 \| . \| .000 \| \| N \| 152 \| 152 \| 152 \| \| hsa-miR-335-5p \| Correlation Coefficient \| -.532^**^ \| .531^**^ \| 1.000 \| \| Sig. (2-tailed) \| .000 \| .000 \| . \| \| N \| 152 \| 152 \| 152 \| \| **. Correlation is significant at the 0.01 level (2-tailed). \| \| \| \| \| \|   (B)   \| **Correlations** \| \| \| \| \| \| \| --- \| --- \| --- \| --- \| --- \| --- \| \|  \| \| \| lnc-HNRNPUL2 \| SFTPD mRNA \| hsa-miR-335-5p \| \| Spearman's rho \| lnc-HNRNPUL2 \| Correlation Coefficient \| 1.000 \| -.535^**^ \| -.484^**^ \| \| Sig. (2-tailed) \| . \| .000 \| .000 \| \| N \| 111 \| 111 \| 111 \| \| SFTPD mRNA \| Correlation Coefficient \| -.535^**^ \| 1.000 \| .535^**^ \| \| Sig. (2-tailed) \| .000 \| . \| .000 \| \| N \| 111 \| 111 \| 111 \| \| hsa-miR-335-5p \| Correlation Coefficient \| -.484^**^ \| .535^**^ \| 1.000 \| \| Sig. (2-tailed) \| .000 \| .000 \| . \| \| N \| 111 \| 111 \| 111 \| \| **. Correlation is significant at the 0.01 level (2-tailed). \| \| \| \| \| \|   (C)   \| **Correlations** \| \| \| \| \| \| \| --- \| --- \| --- \| --- \| --- \| --- \| \|  \| \| \| lnc-HNRNPUL2 \| SFTPD mRNA \| hsa-miR-335-5p \| \| Spearman's rho \| lnc-HNRNPUL2 \| Correlation Coefficient \| 1.000 \| -.271^*^ \| -.202 \| \| Sig. (2-tailed) \| . \| .022 \| .092 \| \| N \| 71 \| 71 \| 71 \| \| SFTPD mRNA \| Correlation Coefficient \| -.271^*^ \| 1.000 \| .355^**^ \| \| Sig. (2-tailed) \| .022 \| . \| .002 \| \| N \| 71 \| 71 \| 71 \| \| hsa-miR-335-5p \| Correlation Coefficient \| -.202 \| .355^**^ \| 1.000 \| \| Sig. (2-tailed) \| .092 \| .002 \| . \| \| N \| 71 \| 71 \| 71 \| \| *. Correlation is significant at the 0.05 level (2-tailed). \| \| \| \| \| \| \| **. Correlation is significant at the 0.01 level (2-tailed). \| \| \| \| \| \|   (D)   \| **Correlations** \| \| \| \| \| \| \| --- \| --- \| --- \| --- \| --- \| --- \| \|  \| \| \| lnc-HNRNPUL2 \| SFTPD mRNA \| hsa-miR-335-5p \| \| Spearman's rho \| lnc-HNRNPUL2 \| Correlation Coefficient \| 1.000 \| -.140 \| -.109 \| \| Sig. (2-tailed) \| . \| .214 \| .335 \| \| N \| 81 \| 81 \| 81 \| \| SFTPD mRNA \| Correlation Coefficient \| -.140 \| 1.000 \| .167 \| \| Sig. (2-tailed) \| .214 \| . \| .136 \| \| N \| 81 \| 81 \| 81 \| \| hsa-miR-335-5p \| Correlation Coefficient \| -.109 \| .167 \| 1.000 \| \| Sig. (2-tailed) \| .335 \| .136 \| . \| \| N \| 81 \| 81 \| 81 \| |
| --- | --- | --- | --- | --- | --- | --- | --- | --- | --- | --- | --- | --- | --- | --- | --- | --- | --- | --- | --- | --- | --- | --- | --- | --- | --- | --- | --- | --- | --- | --- | --- | --- | --- | --- | --- | --- | --- | --- | --- | --- | --- | --- | --- | --- | --- | --- | --- | --- | --- | --- | --- | --- | --- | --- | --- | --- | --- | --- | --- | --- | --- | --- | --- | --- | --- | --- | --- | --- | --- | --- | --- | --- | --- | --- | --- | --- | --- | --- | --- | --- | --- | --- | --- | --- | --- | --- | --- | --- | --- | --- | --- | --- | --- | --- | --- | --- | --- | --- | --- | --- | --- | --- | --- | --- | --- | --- | --- | --- | --- | --- | --- | --- | --- | --- | --- | --- | --- | --- | --- | --- | --- | --- | --- | --- | --- | --- | --- | --- | --- | --- | --- | --- | --- | --- | --- | --- | --- | --- | --- | --- | --- | --- | --- | --- | --- | --- | --- | --- | --- | --- | --- | --- | --- | --- | --- | --- | --- | --- | --- | --- | --- | --- | --- | --- | --- | --- | --- | --- | --- | --- | --- | --- | --- | --- | --- | --- | --- | --- | --- | --- | --- | --- | --- | --- | --- | --- | --- | --- | --- | --- | --- | --- | --- | --- | --- | --- | --- | --- | --- | --- | --- | --- | --- | --- | --- | --- | --- | --- | --- | --- | --- | --- | --- | --- | --- | --- | --- | --- | --- | --- | --- | --- | --- | --- | --- | --- | --- | --- | --- | --- | --- | --- |

**Table S6.: Association of serum biomarkers with case of metastasis, relapse, and cancer stage.**

| Group | lnc-HNRNPUL2 | *SFTPD* mRNA | hsa-miR-335-5p | TGF-β | CEA | CA19.9 |
| --- | --- | --- | --- | --- | --- | --- |
| Metastasized | 49 ± 17.1 | 1.13 ± 0.26 | 0.04 | 524.8 ± 114.4 | 18.6 ± 9.2 | 18.2 ± 5.3 |
| Not Metastasized | 28.4 ± 5 | 1.65 ± 0.37 | 0.44 ± 0.1 | 942.4 ± 131.3 | 6.4 ± 2.1 | 9.3 ± 2.5 |
| *p*-value | 0.265 | 0.265 | 3.03E-04 | 0.02 | 0.21 | 0.15 |
| Early stage | 8.9 ± 5.4 | 1.54 ± 0.22 | 0.44 ± 0.16 | 1032.5 ± 242.6 | 10.95 ± 5.4 | 5.31 ± 0.6 |
| Late stage | 42.2 ± 6.6 | 1.6 ± 0.45 | 0.35 ± 0.1 | 797.8 ± 119.9 | 7.4 ± 2.5 | 13.4 ± 3.2 |
| *p*-value | 1.99E-04 | 0.93 | 0.615 | 0.39 | 0.56 | 0.018 |
| *Relapse* | 2.3 ± 32.4 | 0.96 ± 0.91 | 0.05 ± 0.75 | 57 ± 506 | 3.5 ± 2.5 | 6 ± 13.8 |
| *Not-relapsed* | 3.2 ± 51.9 | 0.96 ± 3.4 | 0.06 ± 0.84 | 45 ± 168 | 11.1 ± 2 | 4 ± 23.9 |
| *p-value* | 0.87 | 0.76 | 0.755 | 0.27 | 0.448 | 0.396 |

**Table S7.: Cox Proportional Hazards Analysis for Metastasis-Free Interval (14-month follow-up)**

| **Variable** | **HR** | **95% CI** | **P-value** | **Interpretation** |
| --- | --- | --- | --- | --- |
| **lnc-HNRNPUL2 (high vs. low)** | 1.62 | 0.75 - 3.52 | 0.218 | Not significant |
| ***SFTPD* mRNA (low vs. high)** | 1.38 | 0.64 - 2.98 | 0.408 | Not significant |
| **hsa-miR-335-5p (low vs. high)*** | 3.85 | 1.42 - 10.45 | **0.008** | Significant risk factor |
| **TGF-β (high vs. low)** | 2.01 | 0.93 - 4.34 | 0.075 | Trend toward significance |
| **Stage (III/IV vs. 0/I/II)** | 4.15 | 1.92 - 8.97 | **<0.001** | Significant |
| **Age (>50 vs. ≤50)** | 1.15 | 0.53 - 2.49 | 0.724 |  |
| **Multivariate Model†** | HR = 3.12 | 1.08 - 9.01 | **0.035** | hsa-miR-335-5p remains significant |

- *Cut-offs: median expression; †Adjusted for age and stage

**Supplementary Figures**

**Figure S1. The proof of the expression of the Surfactant protein D (SFTPD) gene in the CRC by Expression ATLAS database (available at** <https://www.ebi.ac.uk/gxa/home>)**.** The strength of expression was reflected by the transcript per million TPM expression unit and was represented by color change whereas the intense blue color represents high expression levels, the blue color represents medium expression, and the light blue color represents low expression.


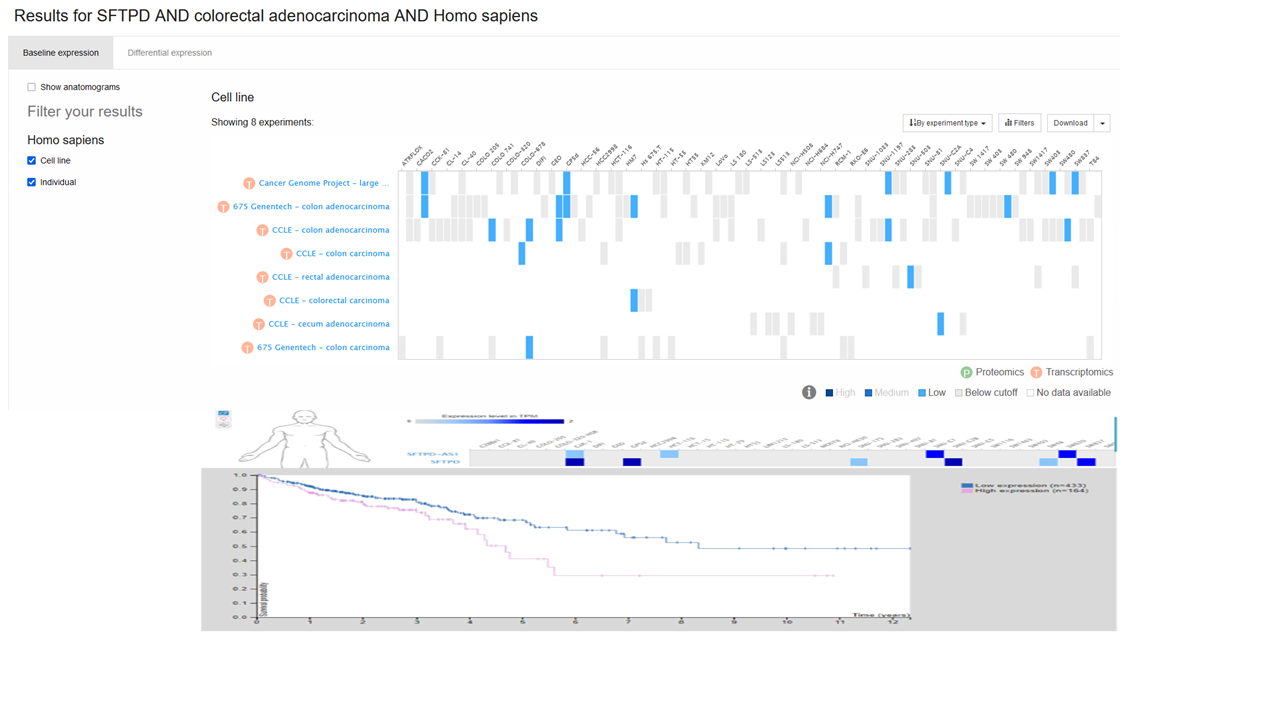


**Figure S2.: Surfactant protein D (SFTPD) gene ontology and expression from the UniProt database (available at** <https://www.uniprot.org/>)**.**


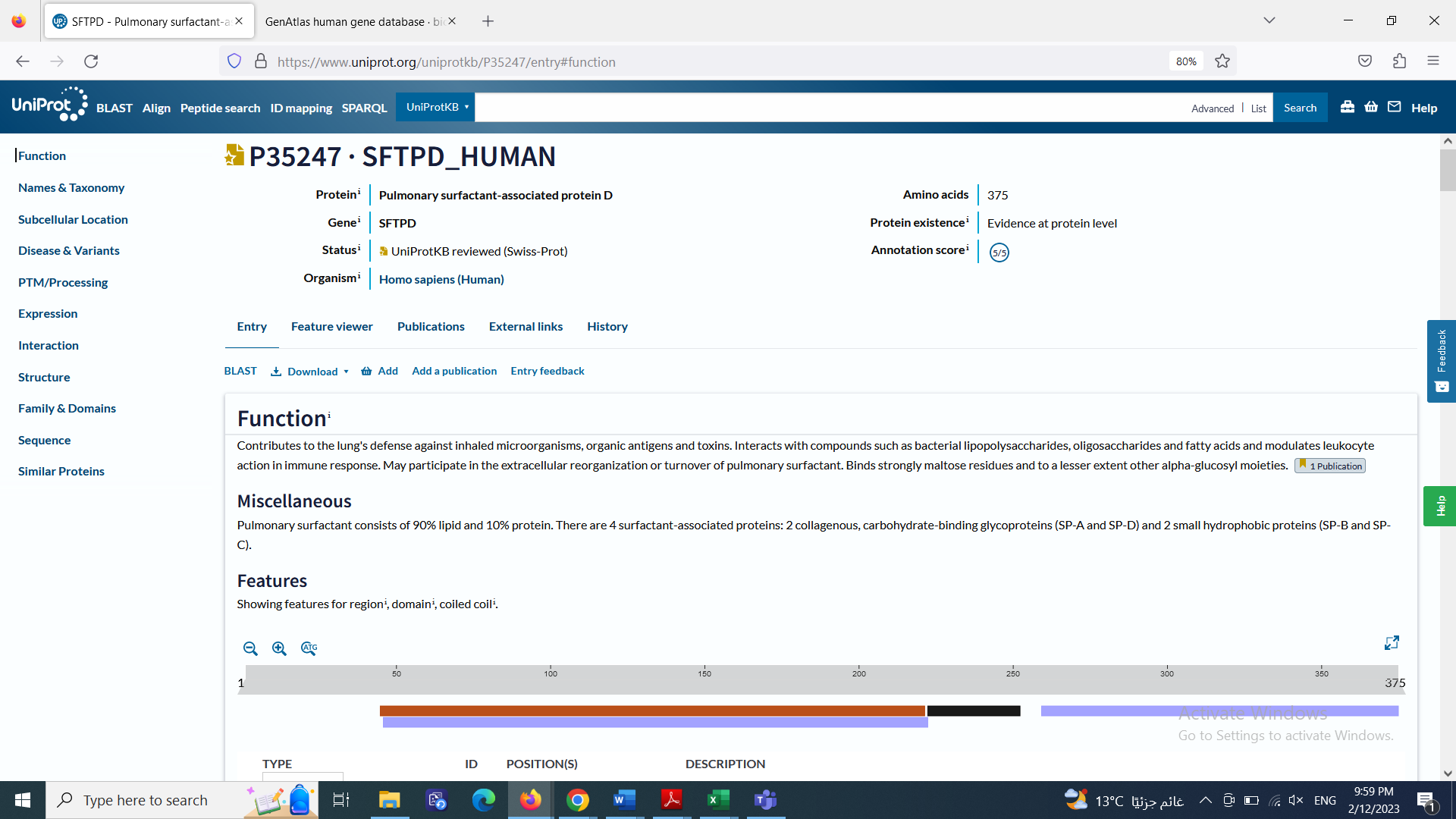


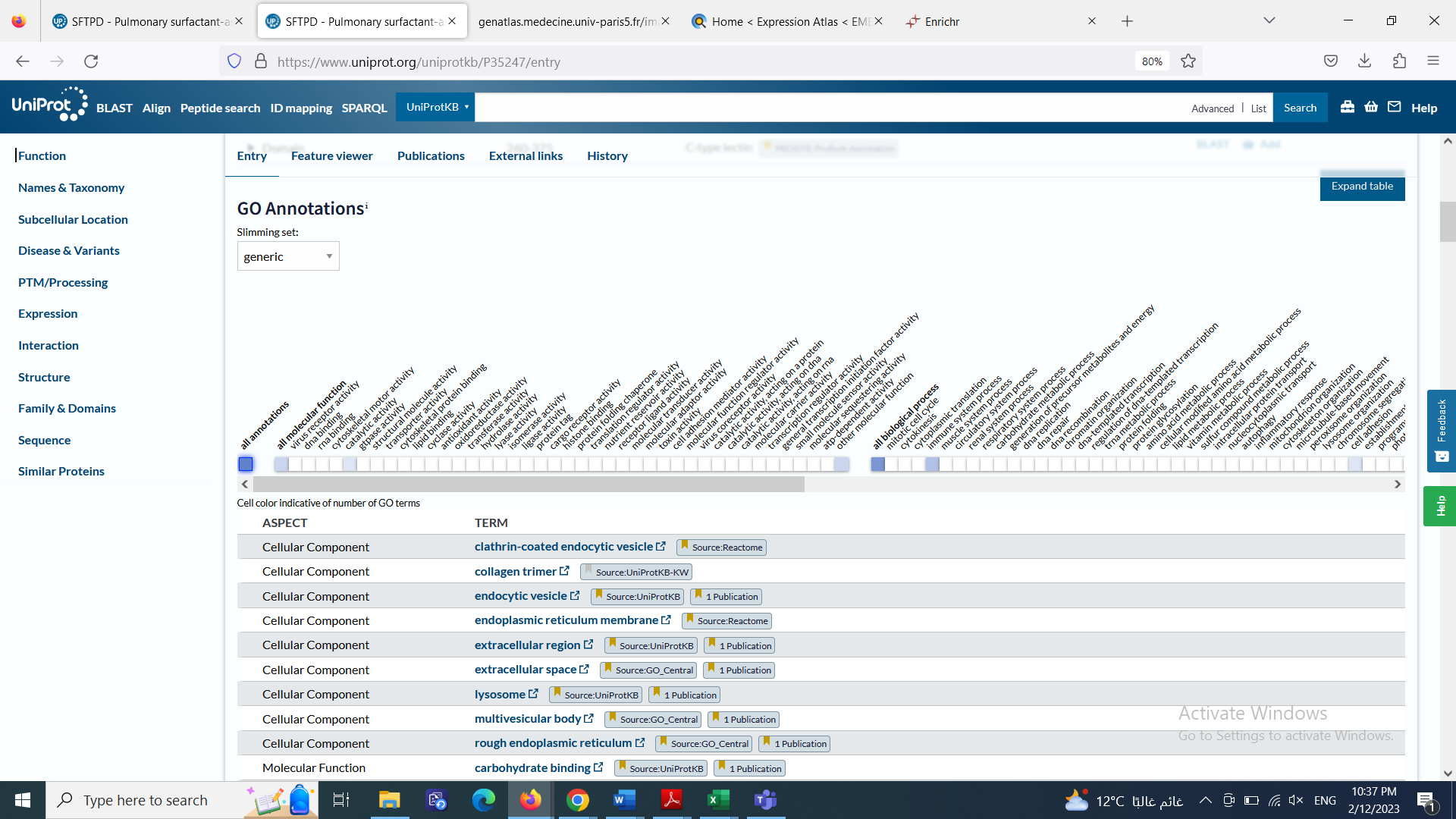


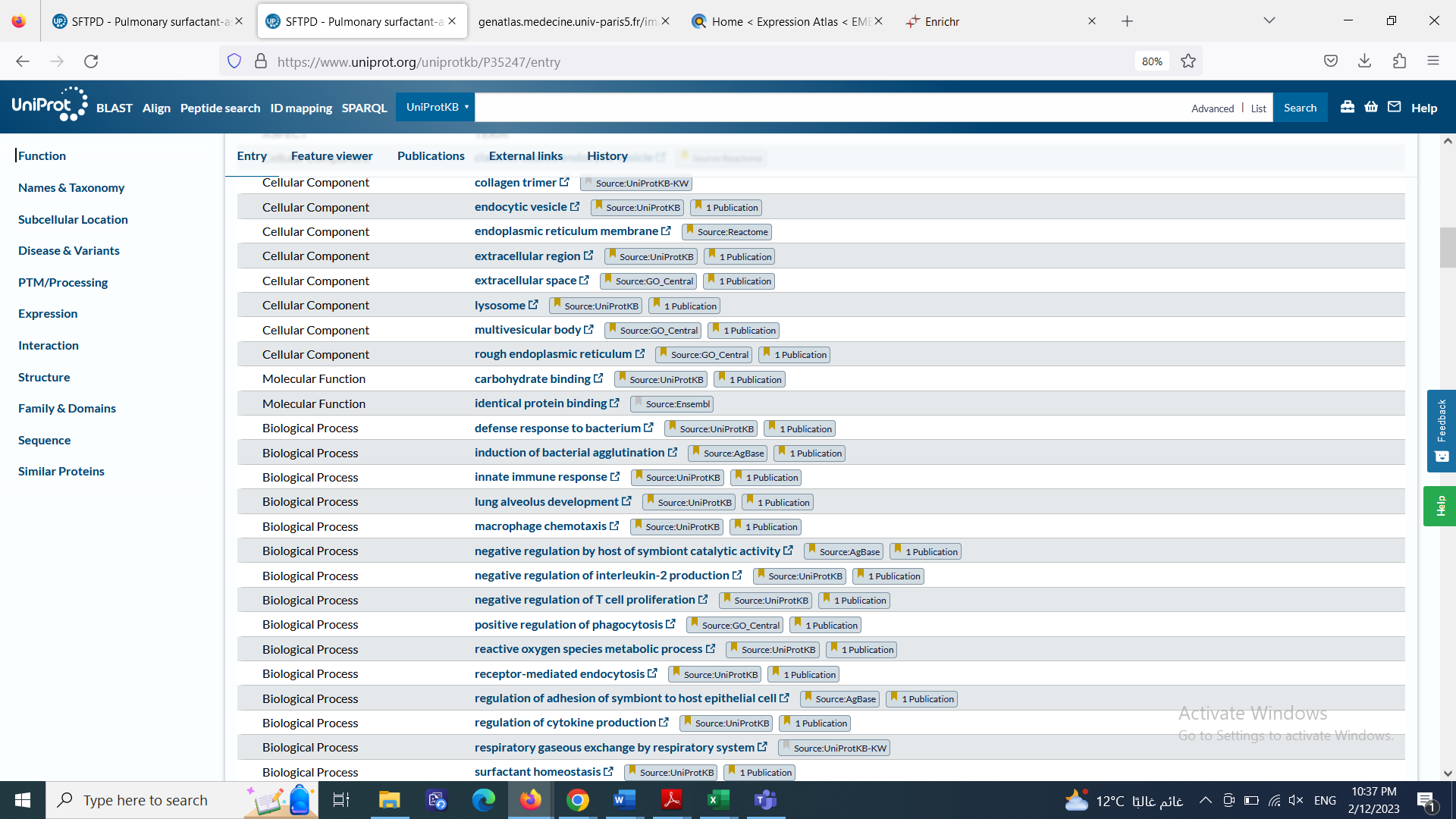


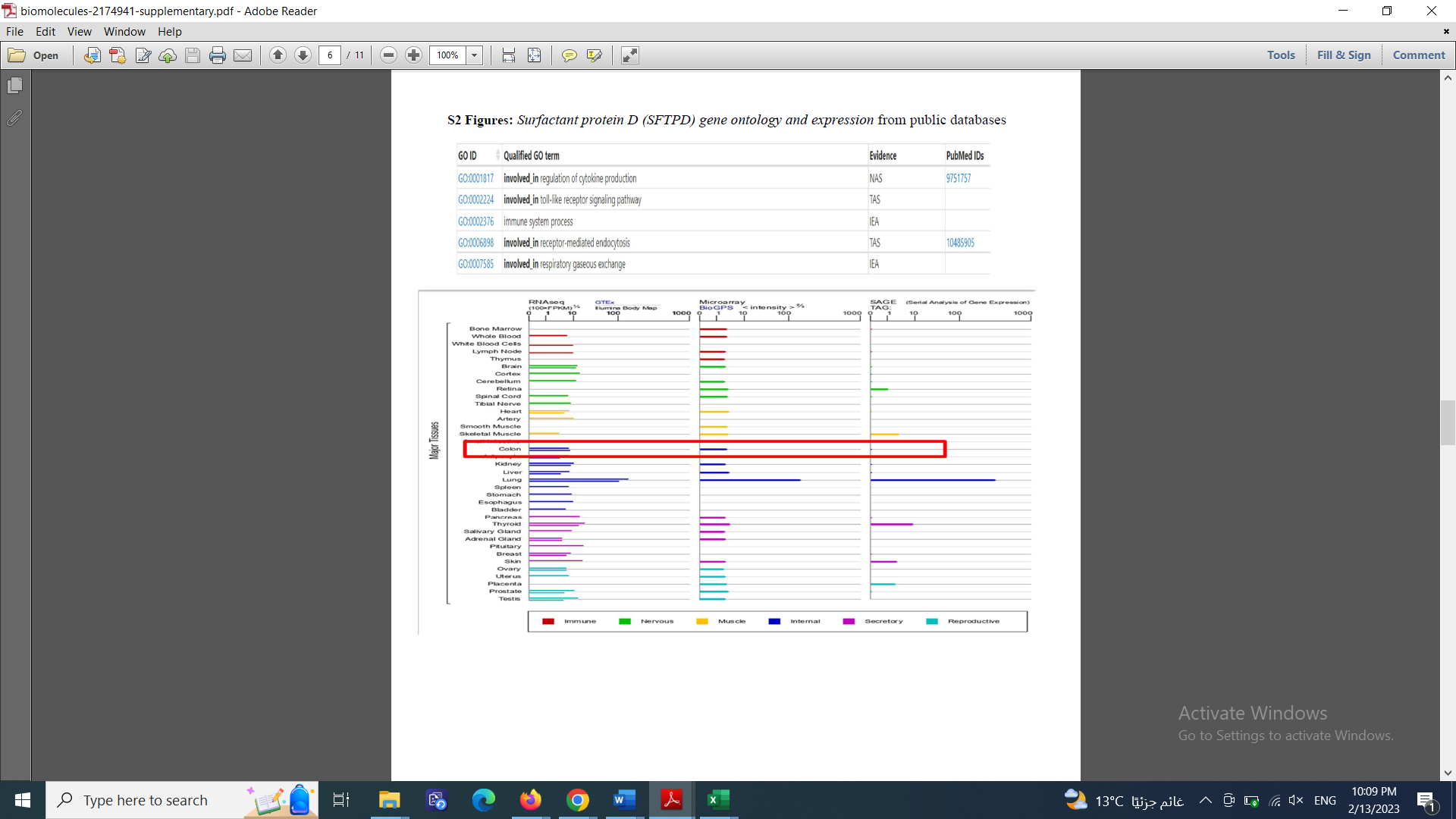


**Figure S3. Represents the interaction between the selected SFTPD mRNA and the has-miR-335-5p in 3 different databases;** miRBase (<https://www.mirbase.org/>), The DIANA database (<https://dianalab.e-ce.uth.gr/html/diana/web/index.php?r=tarbasev8/index>), and miRTarBase database (<https://mirtarbase.cuhk.edu.cn/~miRTarBase/miRTarBase_2022/php/index.php>)**.**


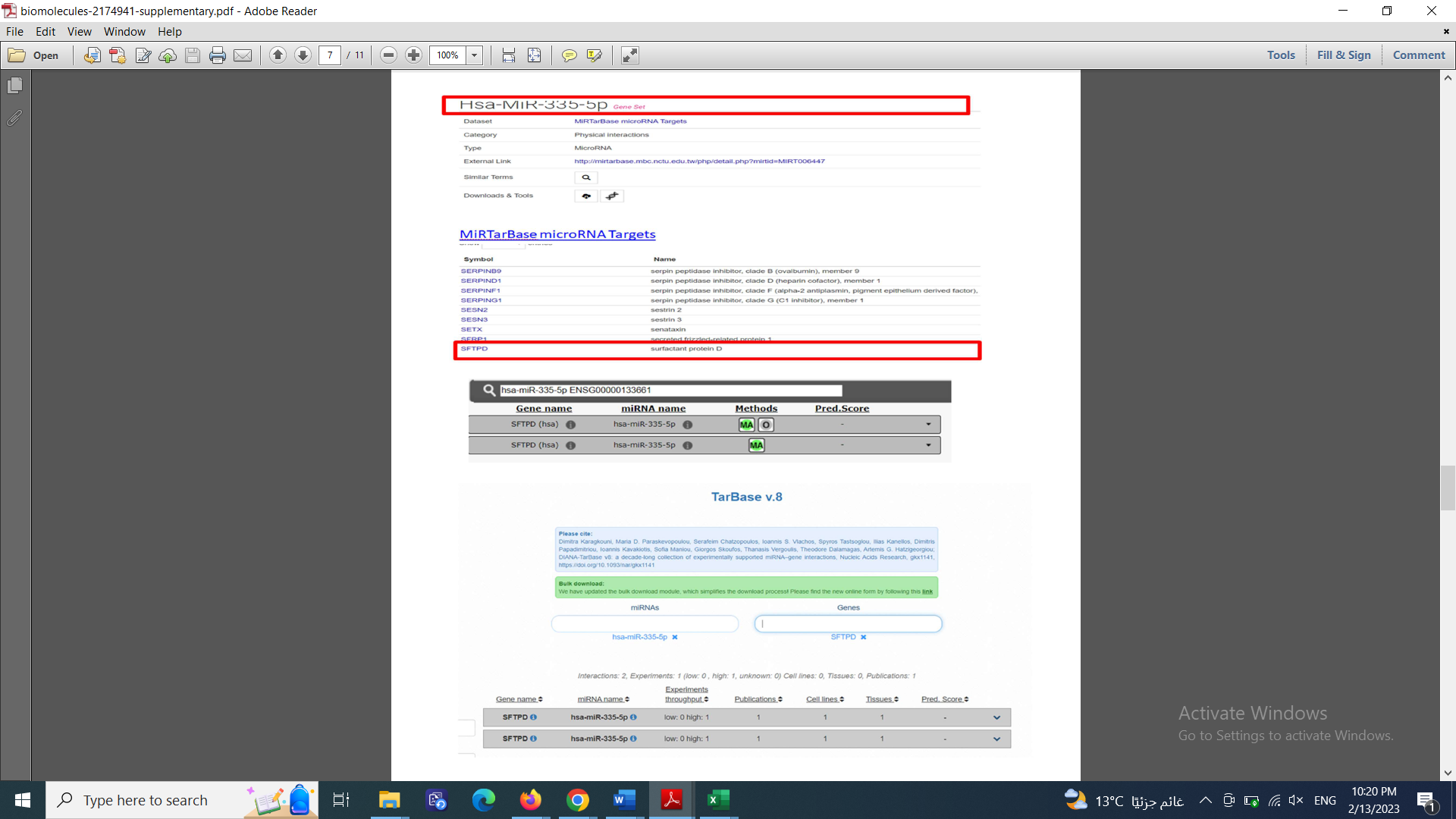


**Figure S4. KEGG pathway analysis for hsa-miR-335-5p by Diana Tools mirPath (**[**https://dianalab.e-ce.uth.gr/html/mirpathv3/index.php?r=mirpath**](https://dianalab.e-ce.uth.gr/html/mirpathv3/index.php?r=mirpath)**).**


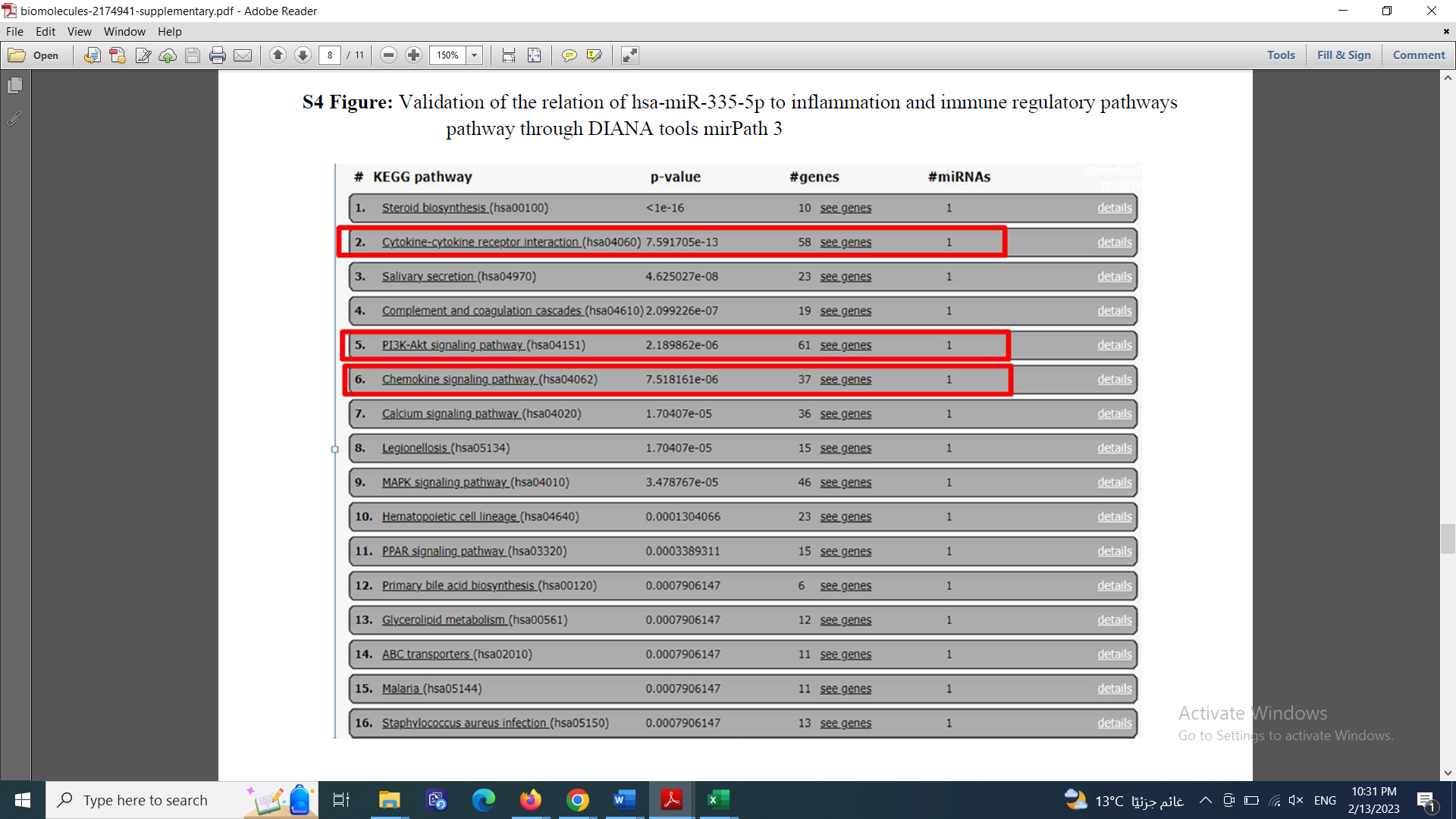


**Figure S5.: The expression of hsa-miR-335-5p in colon tissue by GeneCard Database (**[**https://www.genecards.org/**](https://www.genecards.org/)**).**


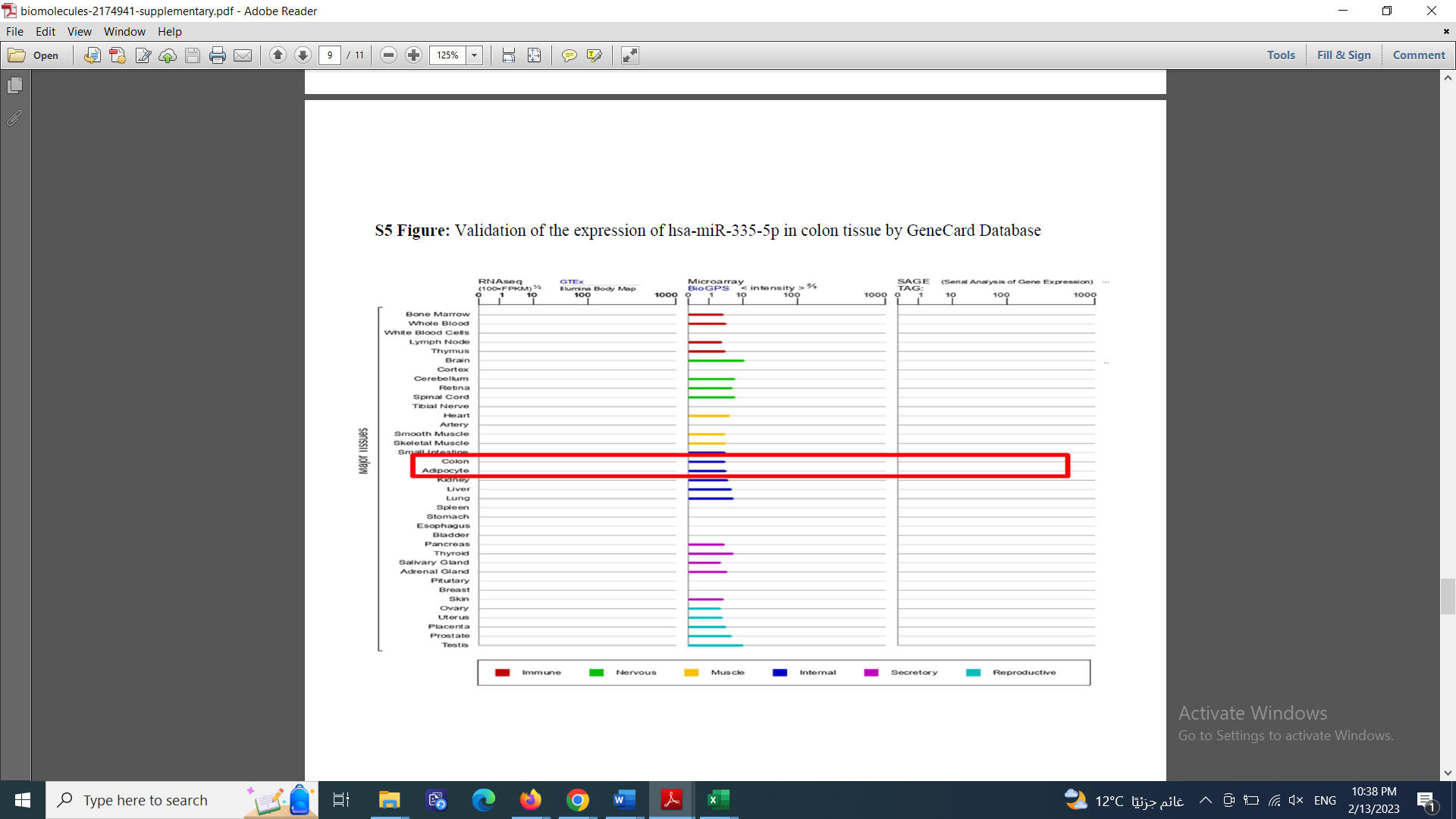


**Figure S6. The interaction between the retrieved lncRNA HNRNPUL2 (NONHSAT021790) and hsa-miR-335-5p using InCeDB database**


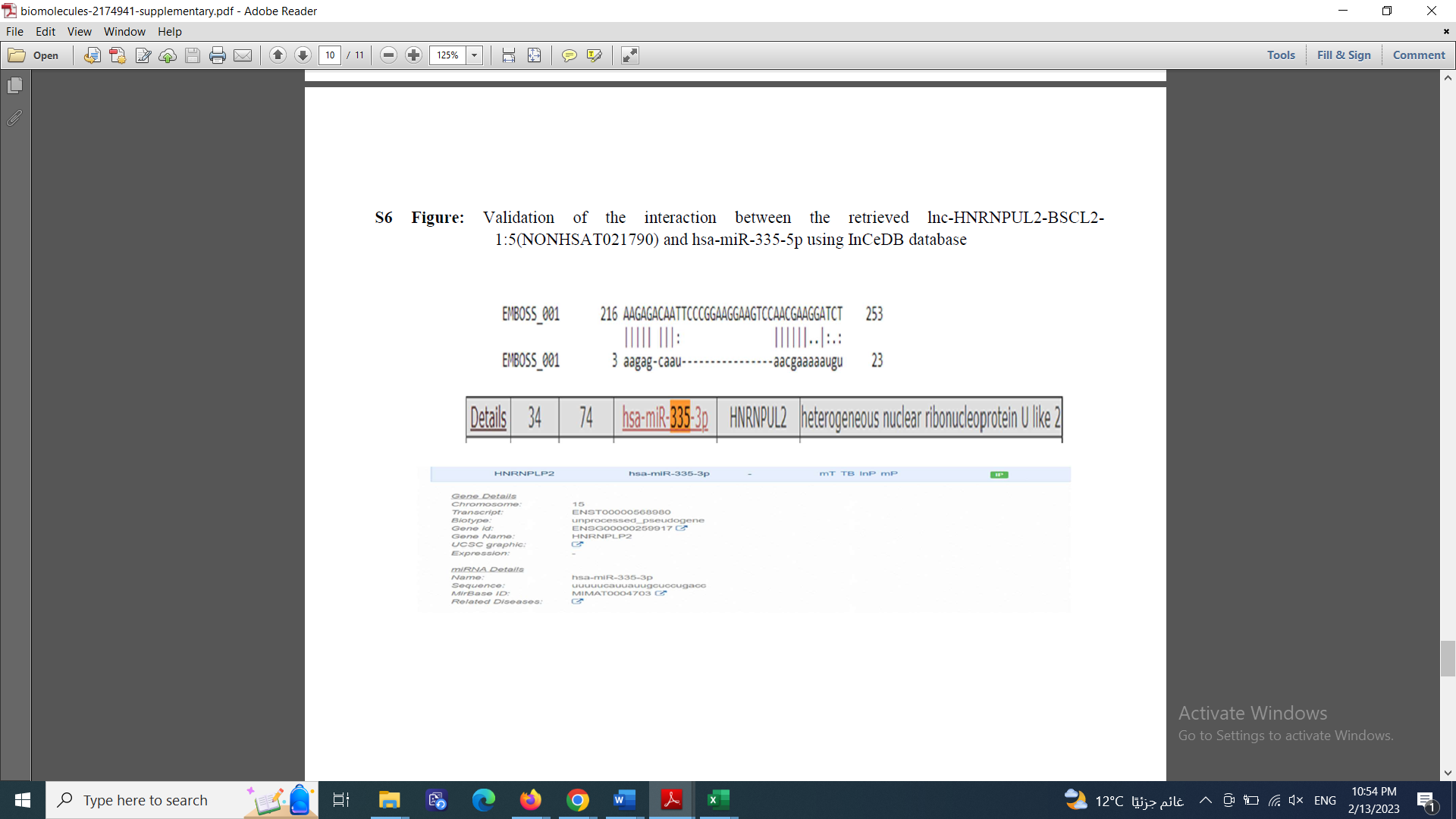


**Figure S7.: The expression HNRNPUL2 in the CRC using the LncBook database (**[**https://ngdc.cncb.ac.cn/lncbook/**](https://ngdc.cncb.ac.cn/lncbook/)**).**


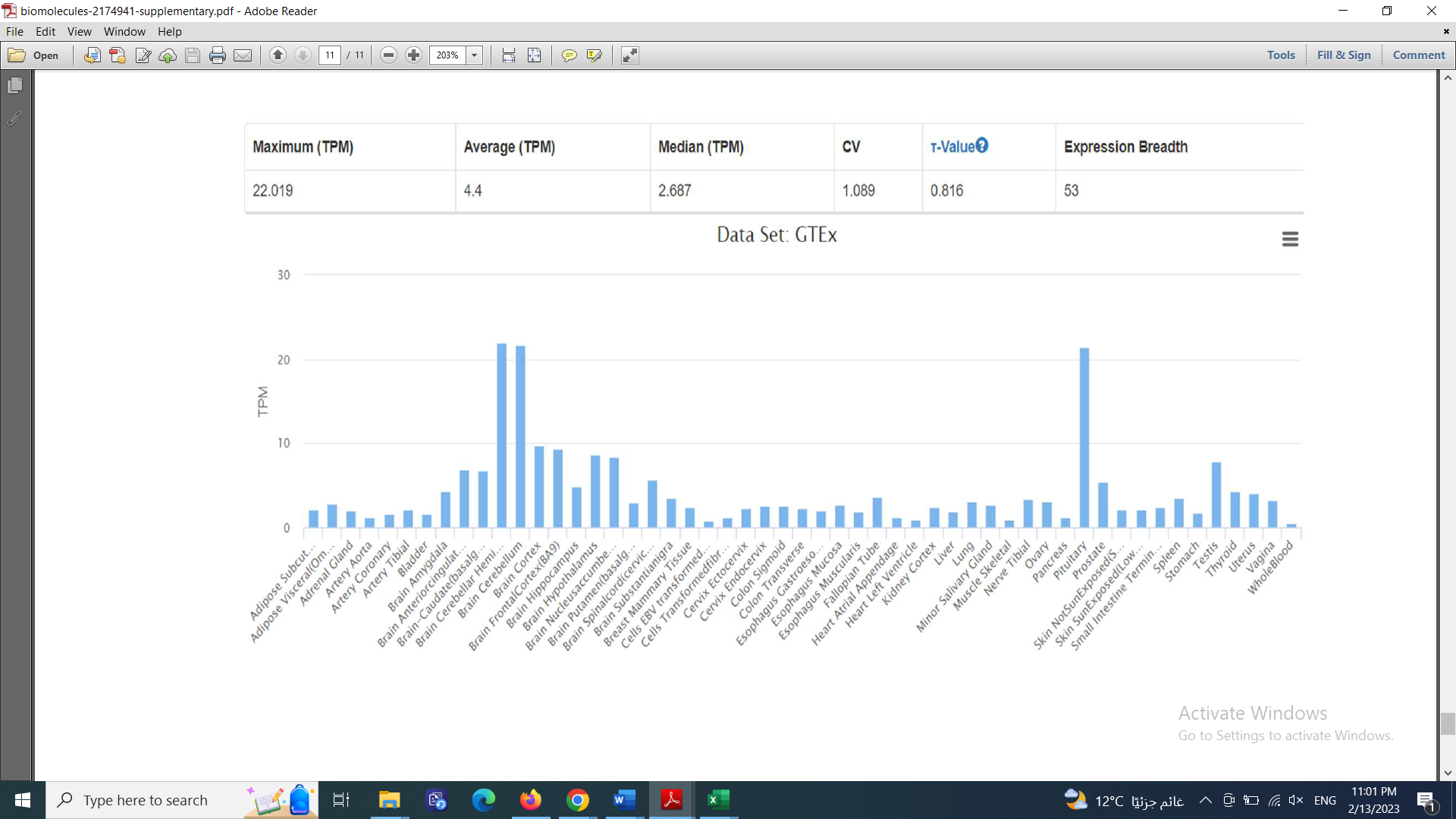


**Figure S8. The interaction between hsa-miR-335-5p and TGF-β using the miRTarBase database (**[**https://mirtarbase.cuhk.edu.cn/~miRTarBase/miRTarBase_2022/php/index.php**](https://mirtarbase.cuhk.edu.cn/~miRTarBase/miRTarBase_2022/php/index.php)**)**


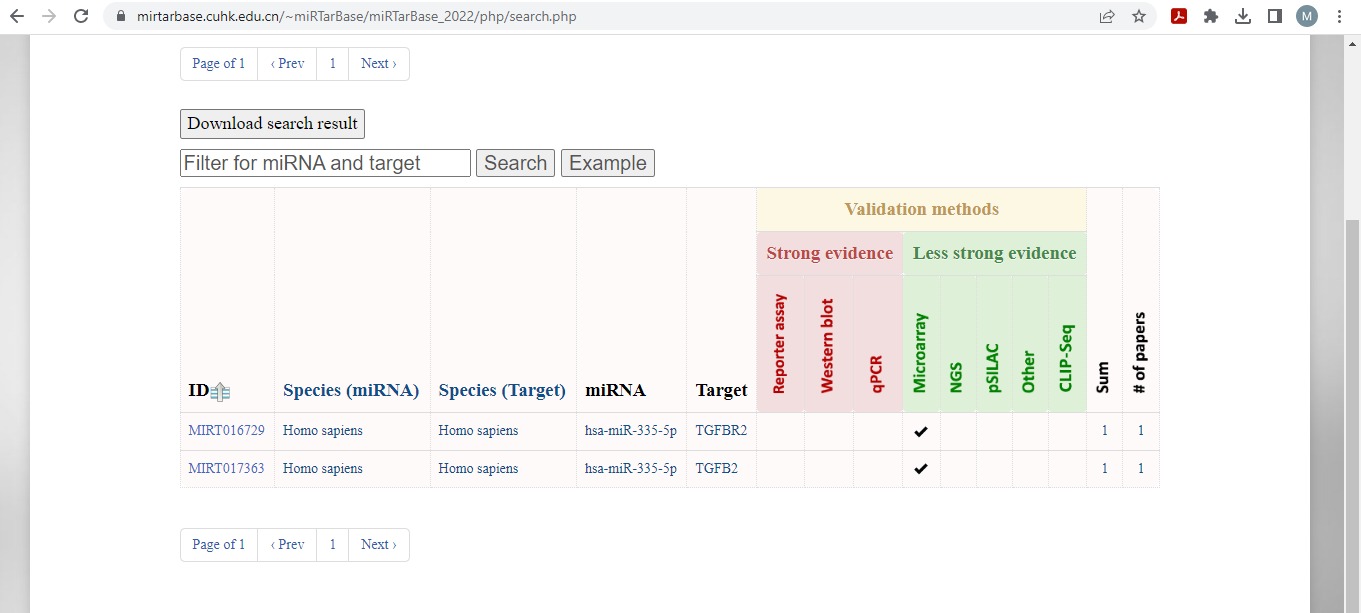


**Figure S9. The interaction between the lnc-HNRNPUL2-BSCL2-1:5 with TGF-β1 and TGF-β2 that was proven by ensemble database (**[**https://www.ensembl.org/index.html**](https://www.ensembl.org/index.html)**)**


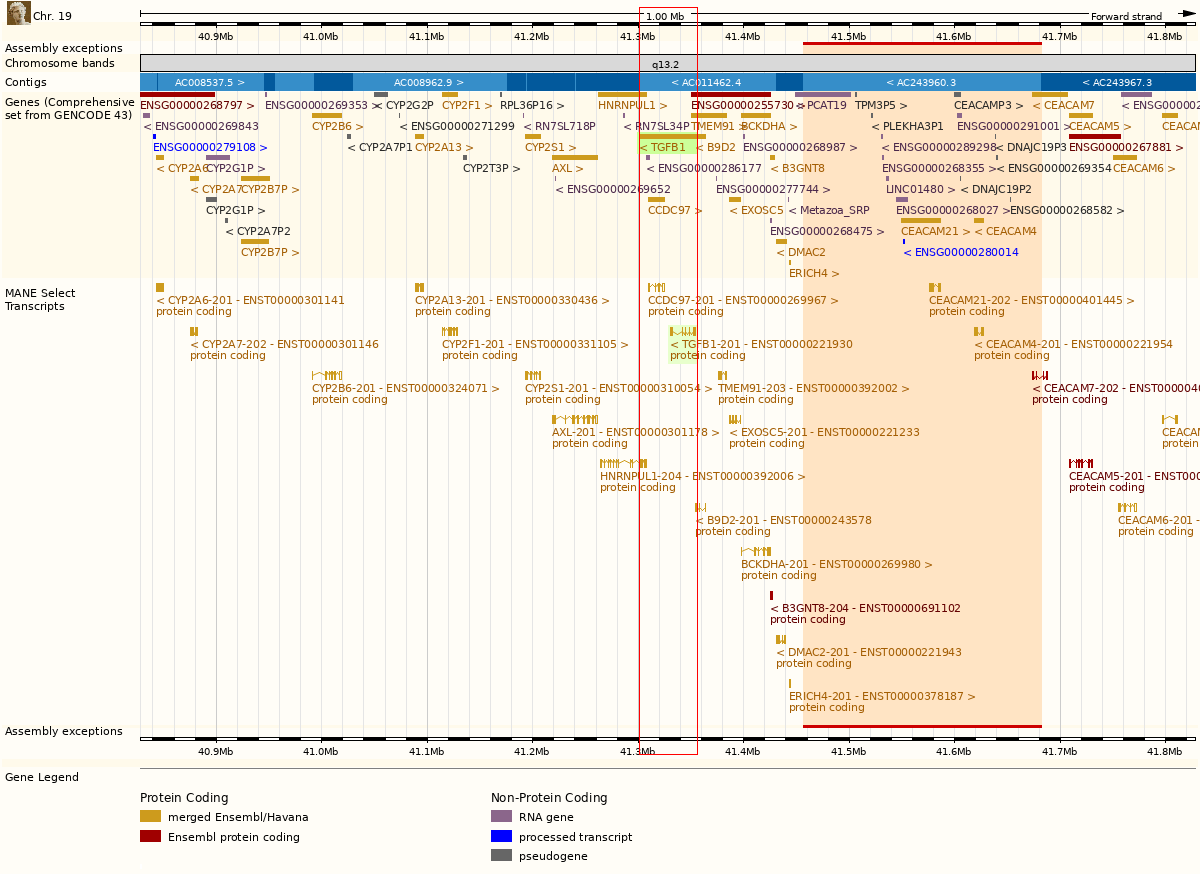


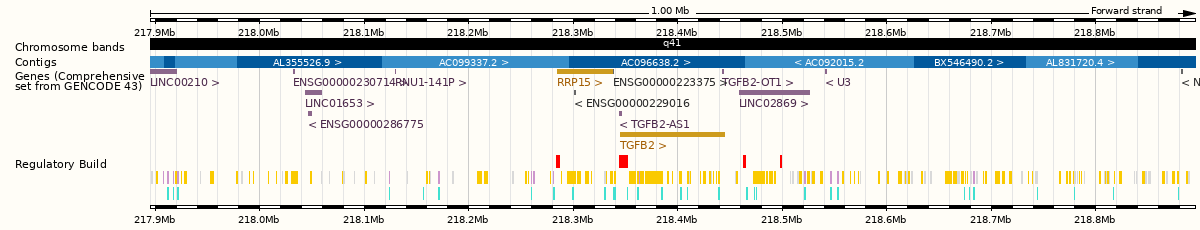


**Figure S10.: Gene ontology matching score for the circulating lncRNA-miRNA-mRNA under study using the Enricher database, including; A: Cellular component. B: Molecular function. C: Biological processes (**[**https://maayanlab.cloud/Enrichr/**](https://maayanlab.cloud/Enrichr/)**).**


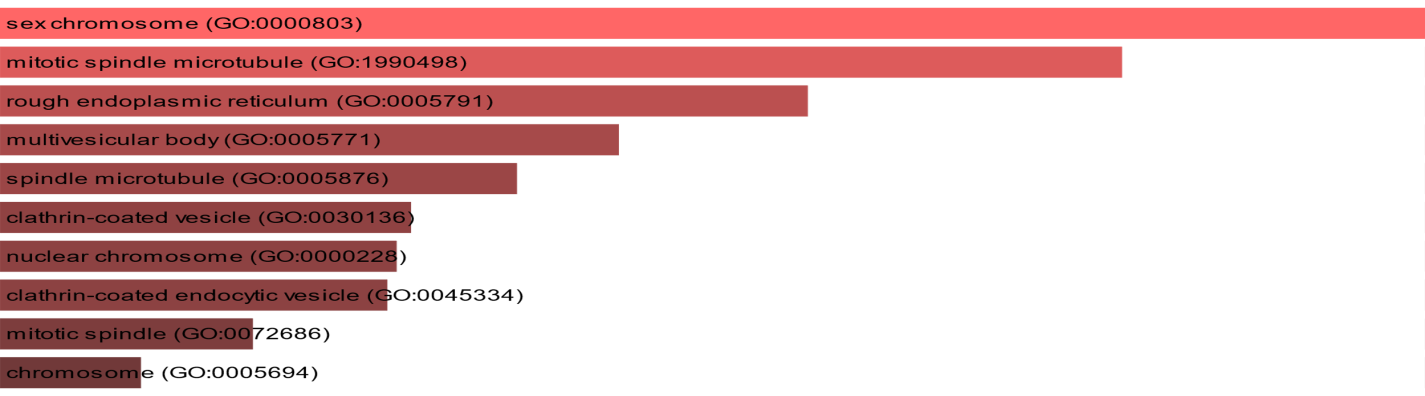


(A) Cellular Component


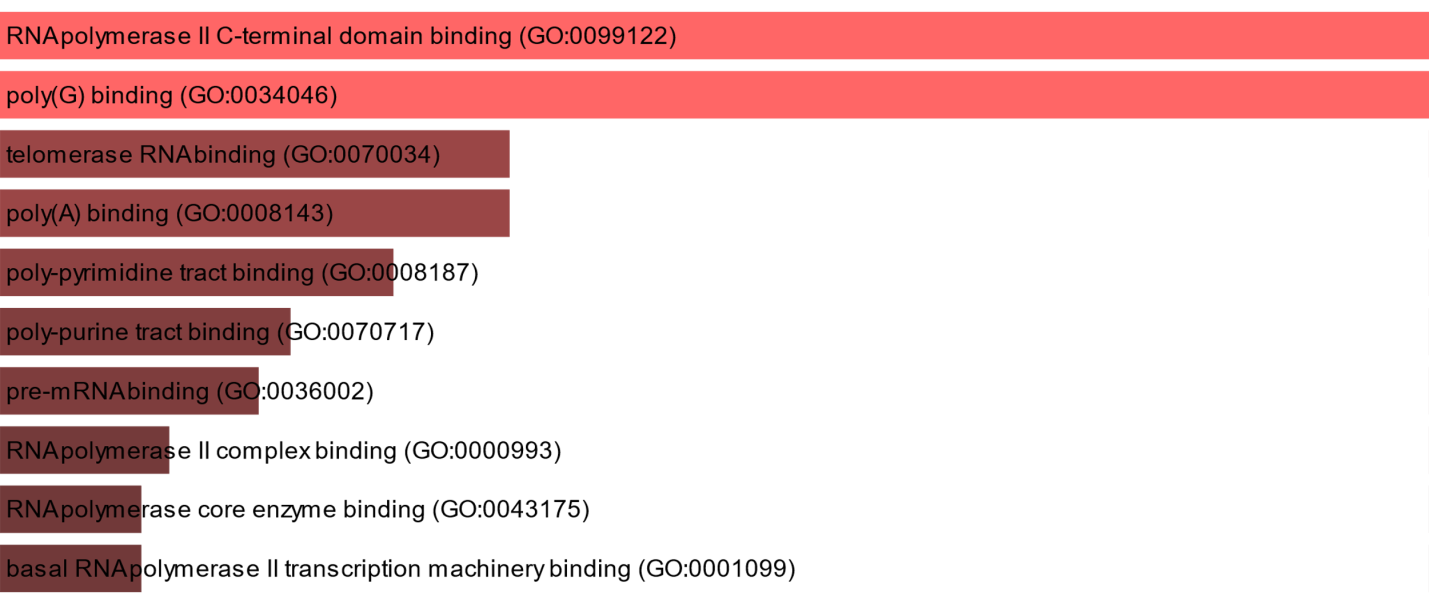


(B) Molecular Function


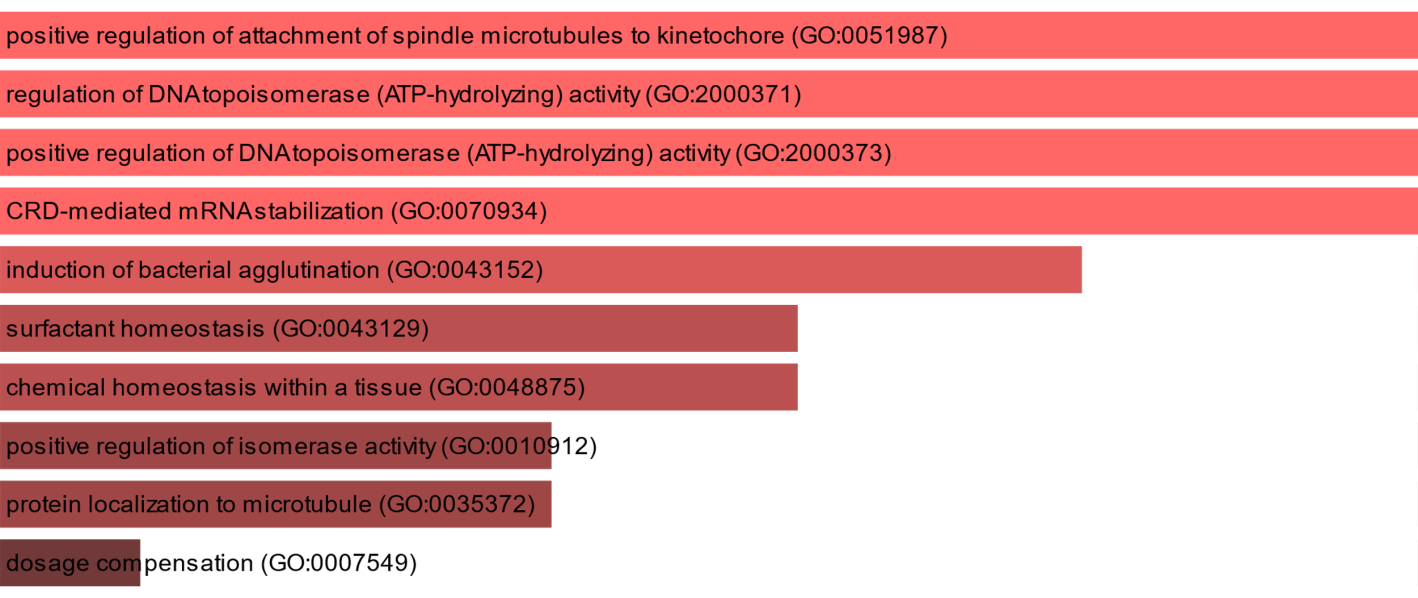


(C) Biological process

**Figure S11.:** **Kaplan–Meier curves for disease-specific survival rate according to the serum levels of (a) lnc-HNRNPUL2, (b) *SFTPD* mRNA, and (c)hsa-miR-335-5p**

| **(a)**  **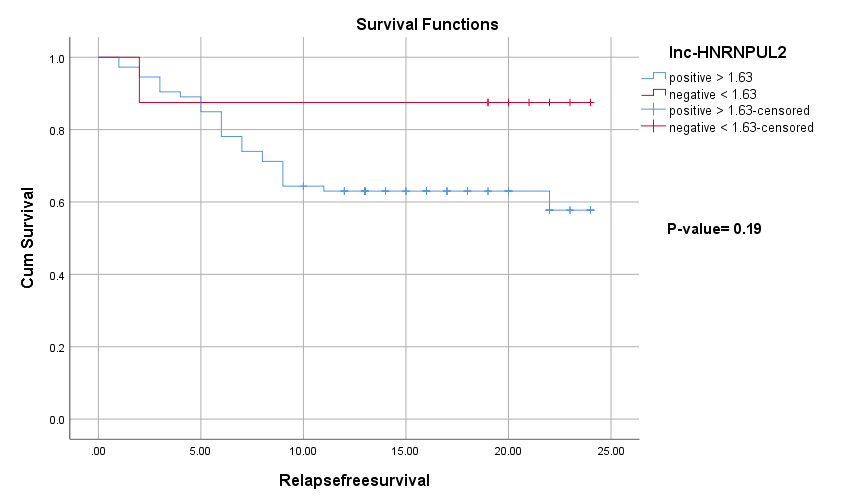**  **(b)**  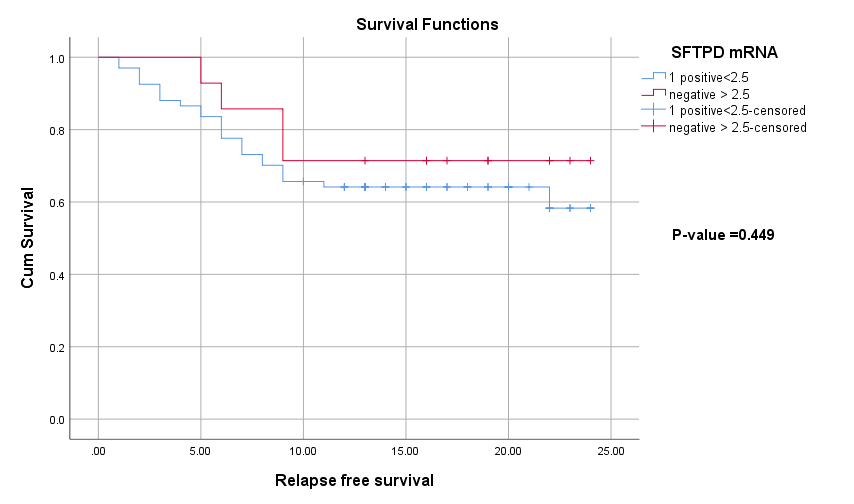  **(c)**  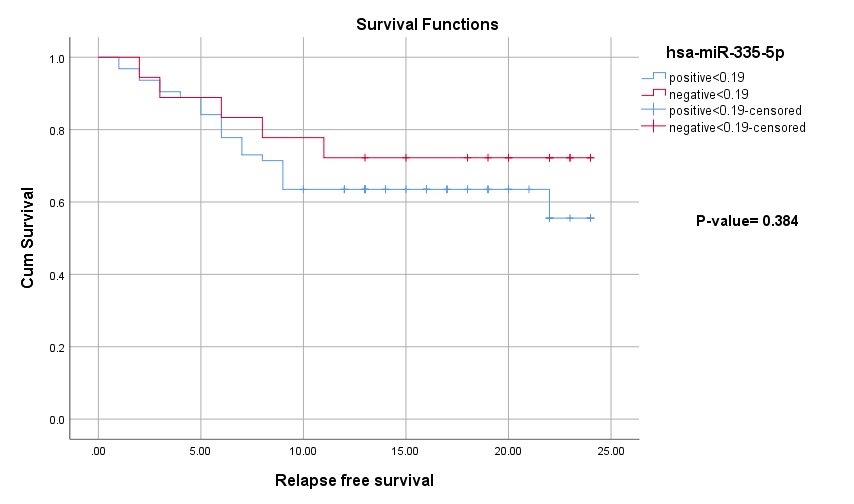 |
| --- |
